# Noninvasive application of mesenchymal stem cell spheres derived from hESC accelerates wound healing in a CXCL12-CXCR4 axis-dependent manner

**DOI:** 10.7150/thno.32982

**Published:** 2019-08-14

**Authors:** Xiaoyan Wang, Bin Jiang, Huiyan Sun, Dejin Zheng, Zhenwu Zhang, Li Yan, Enqin Li, Yaojiong Wu, Ren-He Xu

**Affiliations:** 1 Center of Reproduction, Development & Aging, and Institute of Translational Medicine, Faculty of Health Sciences, University of Macau, Taipa, Macau, China; 2 School of Artificial Intelligence, Jilin University, Changchun, China; 3 The Shenzhen Key Laboratory of Health Sciences and Technology, Graduate School at Shenzhen, Tsinghua University, Shenzhen, China

**Keywords:** Human embryonic stem cells, mesenchymal stem cells, spheroids, wound healing, CXCL12/CXCR4

## Abstract

Mesenchymal stem cells (MSC) derived from adult tissues effectively promote wound healing. However, MSC quality varies, and the quantity of MSC is limited, as MSC are acquired through donations. Moreover, the survival and functioning of dissociated MSC delivered to an inflammatory lesion are subject to challenges.

**Methods**: Here, spheres (EMSC_Sp_) generated from human embryonic stem cell-derived MSC (EMSC) were directly dropped onto excised wounds in mice; the effects of EMSC_Sp_ were compared to those of dissociated EMSC (EMSC_Diss_). Following transplantation, we measured the extent of wound closure, dissected the histological features of the wounds, determined transcriptomic changes in cells isolated from the treated and control wounds, and evaluated the molecular mechanism of the effects of EMSC.

**Results**: The application of EMSC_Sp_ onto murine dermal wounds substantially increased survival and efficacy of EMSC compared to the topical application of EMSC_Diss_. RNA sequencing (RNA-Seq) of cells isolated from the wounds highlighted the involvement of CXCL12-CXCR4 signaling in the effects of EMSC_Sp_, which was verified in EMSC via CXCL12 knockdown and in target cells (vascular endothelial cells, epithelial keratinocytes, and macrophages) via CXCR4 inhibition. Finally, we enhanced the biosafety of EMSC_Sp_ by engineering cells with an inducible suicide gene.

**Conclusions**: Together, these data suggest the topical application of EMSC_Sp_ as an unlimited, quality-assured, safe, and noninvasive therapy for wound healing and the CXCL12-CXCR4 axis as a key player in this treatment.

## Introduction

Wound healing requires cells, cytokines, and matrix proteins to work concertedly. Among these elements, endogenous mesenchymal stem cells (MSC) play a key role in coordinating the repair response by recruiting other cells, cytokines, and matrix proteins 1, 2. MSC can self-renew and differentiate into bone, cartilage, tendons, fat, and many cell types from the mesenchyme 3, 4. In addition, MSC regulate the immune response and inflammation and exert cytoprotective and reparative effects mainly through cell-cell contact and paracrine signaling; thus, MSC are widely used for the treatment of many inflammatory and autoimmune diseases 5, 6.

Exogenous MSC also promote wound healing, as shown in many animal models and clinical trials 7, 8. MSC promote tissue repair via cell empowerment (*e.g.*, recruiting reparative cells, secreting growth factors and matrix proteins, and regulating the immune response and inflammation), cell replacement (*i.e.*, differentiating into cell types to directly participate in tissue repair) or both 9, 10. As a result, MSC can (1) expedite wound closure and re-epithelialization; (2) improve the quality and strength of the regenerated tissue; (3) prevent pathologies that might otherwise lead to a chronic, nonhealing wound; and (4) inhibit scar formation 11. MSC have been isolated from a variety of somatic (including fetal and adult) tissues, such as the umbilical cord, placenta, bone marrow (BM), and adipose tissue 8. However, the supply of somatic tissue-derived MSC relies on donated tissues, the quality of the derived MSC varies, and the transmission of pathogens from donors or contamination during MSC isolation are of great concern.

We reported that MSC derived from human embryonic stem cells (hESC), EMSC, had substantial effects on the treatment of a murine model of experimental autoimmune encephalomyelitis (EAE) 12. Furthermore, we identified a novel approach to the efficient differentiation of hESC into MSC via an intermediate trophoblast-like step and demonstrated that EMSC are efficacious for the treatment of murine models of EAE and chemically induced colitis 13. Unlike somatic tissue-derived MSC, a stable and unlimited supply of EMSC can be derived from a single hESC line; thus, EMSC can be developed as a potential cell therapy to treat autoimmune and inflammatory diseases 6. Because MSC express low levels of human leukocyte antigens (HLAs) (which increase in response to inflammatory signals), inhibit the immune response and do not require long-term engraftment to exert their effects, allogeneic and even xenogeneic MSC are often as efficacious as autologous MSC 9.

Current methods for MSC delivery, including intravenous (i.v.) injection for systematic distribution and local injection into target tissues, are often invasive 14. Furthermore, cell viability following injection is unclear. Additional methods have been seeking to increase the viability and efficacy of MSC and reduce inconvenience to clinicians and patients. Recently, we found that the formation of EMSC spheres (EMSC_Sp_) allows the cells to survive ambient conditions in a sealed tube for up to 10 days by reducing cell metabolism and proliferation to endure the harsh environment. We thoroughly characterized EMSC_Sp_ in a previous study. Specifically, each sphere was formed from 2 × 10^4^ EMSC. The spheres were approximately 400 µm in size, and there were no necrotic cells in the center of the spheroids 15.

Based on these data, we hypothesized that EMSC_Sp_ transplanted to a skin wound can tolerate the superficial and inflammatory environment and that the survival rate and function of EMSC_Sp_ are greater than those of dissociated EMSC (EMSC_Diss_). We employed a murine model of excisional splinted skin wounds, in which contraction of the skin around a wound is prevented so the wound healing effect of a treatment can be displayed and measured objectively 16. We directly dropped EMSC_Sp_ onto the wound surface, which promoted much faster wound healing than that following the local delivery of EMSC_Diss_. Similar results were observed when the effects of MSC spheres derived from human bone marrow (BM-MSC_Sp_) were compared to those of dissociated control cells. Finally, to reduce tumorigenesis concerns, we transduced EMSC with chemically inducible caspase-9 (iCasp9) 17 and demonstrated that the engineered EMSC can be efficiently killed *in vitro* and eliminated *in vivo* after therapy.

## Results

### Accelerated wound closure by EMSC_Sp_

**Figure 1:**
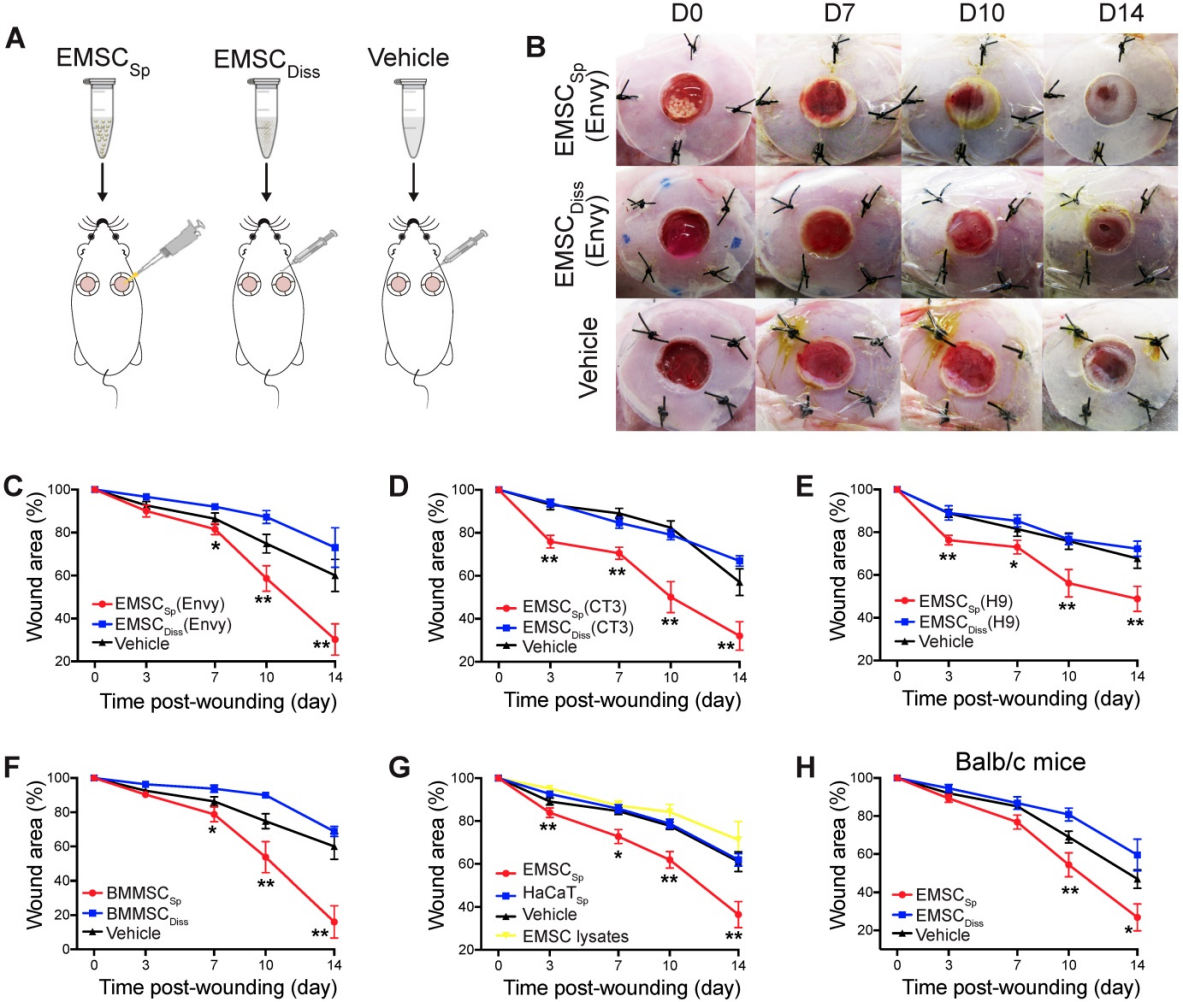
** Effects of EMSC on wound closure.** (A) Experimental scheme for the development of an excisional wound splint model in NOD/SCID mice and the transplantation of EMSC_Sp_, EMSC_Diss_ or the vehicle control. (B) Representative images of wounds in mice treated as above at various time points following wound formation. (C-E) The percentage of the wound area in NOD/SCID mice was measured after the transplantation of EMSCs derived from Envy (C), CT3 (D), and H9 (E) hESC lines. n = 5, 8, and 6 biological repeats in C, D, and E, respectively. *P < 0.05 and **P < 0.01 for EMSC_Sp_ versus EMSC_Diss_ or the vehicle control per ANOVA followed by Tukey's multiple comparison test. (F) The percentage of the wound area was measured in NOD/SCID mice after transplantation of BM-MSC_Sp_, BM-MSC_Diss_ or the vehicle. n = 5 biological repeats; per ANOVA followed by Tukey's test, *P < 0.05 and **P < 0.01 for BM-MSC_Sp_ versus BM-MSC_Diss_ or the vehicle control. (G) The percentage of the wound area was measured in mice after transplantation of EMSC_Sp_, spheres formed by HaCaT cells (HaCaT_Sp_), and EMSC lysates. n = 4 biological repeats, *P < 0.05 and **P < 0.01 for EMSC_Sp_ versus HaCaT_Sp_ or the EMSC lysates per ANOVA analysis. (H) The percentage of the wound area was measured in Balb/c mice after transplantation of EMSC_Sp_, EMSC_Diss_ or the vehicle. n = 6 biological repeats; per ANOVA followed by Tukey's test, *P < 0.05 and ** *P* < 0.01 for EMSC_Sp_ versus EMSC_Diss_.

We first established an excisional splinted skin wound model 16 in immunocompromised NOD/SCID mice, and Envy hESC constitutively expressing green fluorescent protein (GFP) 18 were differentiated into EMSC using previously reported methods 13, 19. EMSC were cultured in a monolayer and passaged weekly, and only EMSC within passage 10 were used in this study. EMSC_Diss_ were prepared by dissociating EMSC in monolayer culture with trypsinization, and EMSC_Sp_ were formed from the dissociated EMSC (Fig. S1A) using the hanging drop method 15. Immediately after wound formation, the mice were randomly divided into three groups: the EMSC_Sp_, EMSC_Diss_, and vehicle control groups (Fig. 1A). For EMSC_Sp_ treatment, 40 spheres, which is equivalent to 1×10^6^ EMSC, were directly dropped onto the surface of each wound. For EMSC_Diss_ treatment, a previously reported combined method with optimal efficacy 16 was used to deliver the dissociated cells; a total of 0.7×10^6^ EMSC_Diss_ in 100 μl PBS (70%) per wound were evenly injected into the dermis in four spots (above, below, left, and right) around the wound, and 0.3×10^6^ EMSC_Diss_ (30%) mixed with 30 μl Matrigel were dropped onto the surface of the wound. For the vehicle control group, 100 μl PBS alone per wound was evenly injected around the wound, and 30 μl Matrigel was dropped onto the surface of the wound as described above (Fig. S1A).

After transplantation, EMSC_Sp_ stayed firmly on the wound and were initially visible through the transparent Tegaderm (Fig. S1B). By day 7 posttreatment, no remarkable differences in wound closure were observed among the three groups. By day 10, wound closure had accelerated in the EMSC_Sp_ group compared to that in the EMSC_Diss_ and vehicle control groups, which became more evident on day 14. Surprisingly, wound closure was even slower in the EMSC_Diss_ group than in the vehicle control group (Fig. 1B and 1C), suggesting that dissociated EMSC did not promote and instead delayed wound healing. Nevertheless, greater success might be achievable with increased doses of EMSC_Diss_ and/or via the administration of EMSC_Diss_ through a different route. Enhanced wound healing was also observed following treatment with EMSC_Sp_ derived from two other hESC lines, CT3 20 and H9 21 cells (Fig. 1D and 1E), and BM-MSC_Sp_, but not dissociated BM-MSC (Fig. 1F), EMSC lysates, and spheres formed by nonMSC such as HaCaT keratinocyte cells, underscoring the importance of live MSC spheres for efficacious treatment (Fig. 1G). In addition to the immunocompromised NOD/SCID mice, immunocompetent mice Balb/c were also treated, and the superior effects of EMSC_Sp_ in promoting wound repair over those of EMSC_Diss_ were observed (Fig. 1H), suggesting that the therapeutic effects of EMSC_Sp_ are not remarkably affected by immunological status. Hereafter, only NOD/SCID mice were used unless otherwise specified.

### Engraftment of EMSC in wounded skin

**Figure 2:**
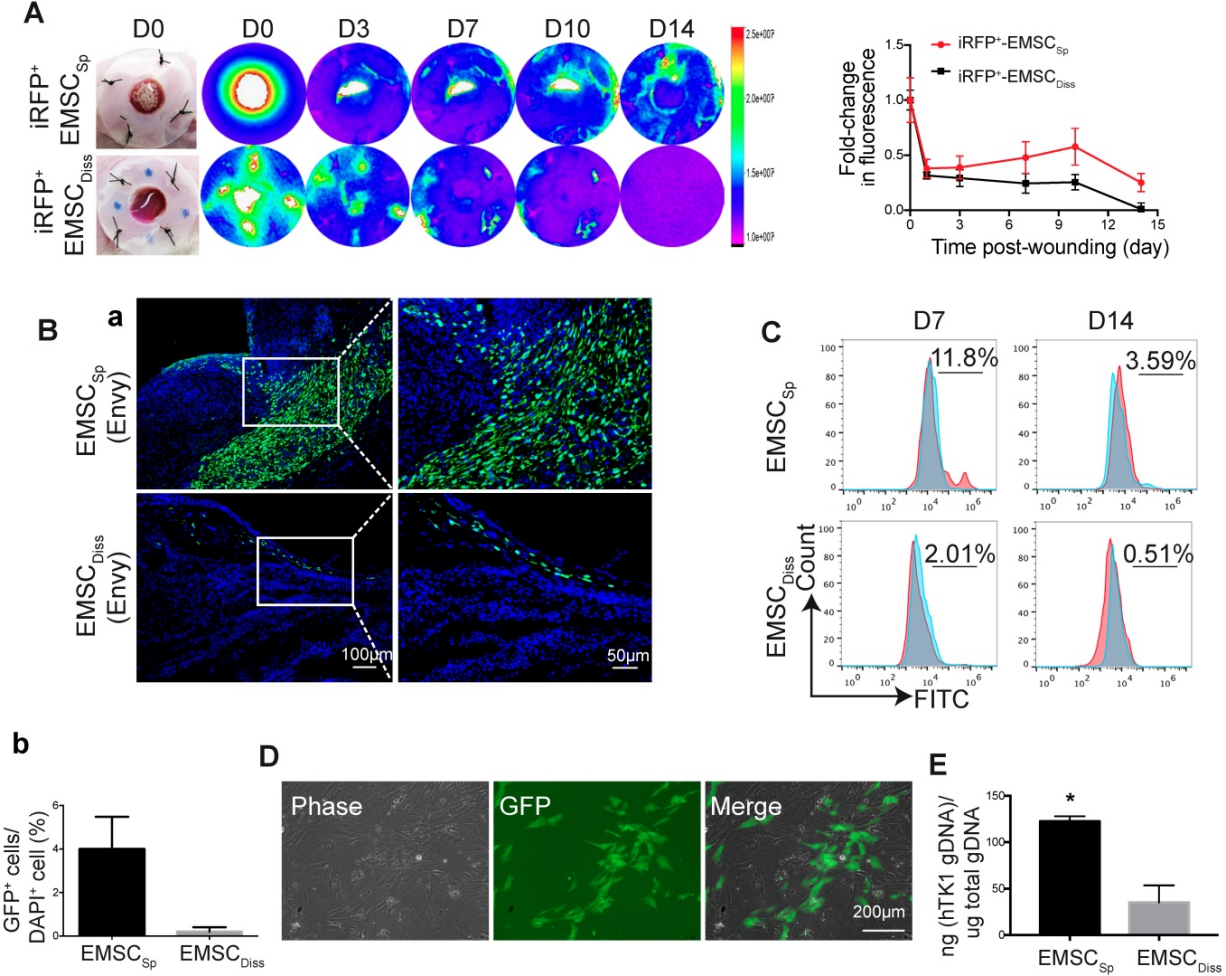
** Viability and engraftment of EMSC following their transplantation to wounds.** (A) Representative bright-field images of wounds with transplanted iRFP-expressing EMSC_Sp_ (upper row) or EMSC_Diss_ (bottom row) were taken on day 0, followed by fluorescent images acquired using the In-Vivo Xtreme System on various days following wound formation. The colored bar indicates the fluorescence intensity. The fold change in the fluorescent intensity is shown in the graph on the right. (B) (a) Immunostaining for GFP^+^ cells in wounds with transplanted Envy hESC-derived EMSC_Sp_ and EMSC_Diss_ 14 days after wound formation. Boxed areas are amplified and shown on the right. (b) The percentage of GFP^+^ cells (determined via DAPI staining of the nucleus) per wound section. (C) Flow cytometry to detect GFP^+^ cells in wounds with transplanted Envy hESC-derived EMSC_Sp_ and EMSC_Diss_ 7 and 14 days after wound formation. Values represent the percentage of GFP^+^ cells from 3 biological repeats. (D) Cultured cells were isolated following the digestion of wounds with transplanted EMSC_Sp_ (Envy) 7 days after wound formation. Bright-field (left) and fluorescent (middle) images of the cultured cells were taken and merged (right). Scale bar = 200 μm. (E) Detection of human *TK1* gDNA in mice 14 days after EMSC transplantation. One x 10^6^ EMSC_Sp_ and EMSC_Diss_ cells were transplanted to the skin wound. After 14 days, wounded skin was dissected, and total gDNA was extracted for PCR to detect human *TK1*. n = 3 biological repeats. * P < 0.05 for EMSC_Sp_ versus EMSC_Diss_ per student *t*-test.

To track transplanted EMSC in live mice, we engineered Envy EMSC to let them to constitutively express a near-infrared fluorescent protein (iRFP) (Fig. S2A) as iRFP is more easily detected *in vivo* and produces less background noise than GFP 22. By using the Bruker In-vivo Xtreme imaging system, we first assured that iRFP signals in the wound area of a mouse were detectable immediately after topical transplantation of the GFP^+^/iRFP^+^ EMSC_Sp_ onto the wound (Fig. S2B). Then, we monitored iRFP signals in the wounds of mice following the local transplantation of EMSC_Sp_ or EMSC_Diss_ as described above. Strong iRFP signals were detected in the wound areas in the mice in both groups immediately after cell delivery. The signals decreased sharply the next day but remained high in the center of the wounds and declined much slower in the EMSC_Sp_-treated mice than in the EMSC_Diss_-treated mice for up to 14 days of observation (Fig. 2A).

To investigate whether topically delivered EMSC infiltrate a healing wound, we treated wounded mice with EMSC_Sp_ or EMSC_Diss_ that were derived from the original Envy hESC line (GFP^+^ only) (Fig. S2C). Following sectioning and immunostaining, many more GFP^+^ cells were found in the wounds of mice treated with EMSC_Sp_ than in the wounds of mice treated with EMSC_Diss_ 14 days after treatment. GFP^+^ cells were mainly located in the center of the wound, especially in the newly formed dermis, some were located in the epidermis, and very few were found in the adjacent normal skin (Fig. 2Ba). The percentage of GFP^+^ cells was much higher in the EMSC_Sp_ group than in the EMSC_Diss_ group (Fig. 2Bb), which was verified through flow cytometry analysis of cells dissociated from the wounds after their dissection from the mice 7 and 14 days after treatment (Fig. 2C). We replated the dissociated cells from both groups. GFP^+^ (EMSC or their derivatives) as well as GFP^-^ (adjacent cells from the host) cells from the EMSC_Sp_ group proliferated well (Fig. 2D), whereas almost no GFP^+^ cells were found in EMSC_Diss-_treated wounds (data not shown). To confirm these findings, we conducted an experiment with EMSC differentiated from mCherry^+^ H9 hESC and observed similar results (Fig. S2D). Meanwhile, the human thymidine kinase-1 (h*TK1)* sequence 23 was detected in human genomic DNA (gDNA) from the wounded skin 14 days after EMSC transplantation, which indicates that many more human cells were engrafted in the EMSC_Sp_ group than in the EMSC_Diss_ group (Fig. 2E).

To determine the viability of cells in the spheres applied onto wounds, we retrieved some spheres from the wound surface at day 4 after implantation. A portion of the spheres was directly replated into Petri dishes to detect GFP^+^ cells (Fig. S2E, bottom left), whereas the remaining spheres were dissociated to test cell viability with an acridine orange (AO)/propidium iodide (PI) kit (Fig. S2E, bottom right). Twenty-four hours later, the replated spheres had attached, and cells had started to migrate away from the attached spheres, with up to 85±5% of the dissociated cells alive per AO/PI staining. These data suggest that more EMSC_Sp_ survived and had been engrafted in the wound area than EMSC_Diss_.

### Histological evidence of EMSC_Sp_-promoted wound healing

Based on the expedited wound closure and improved cell engraftment following EMSC_Sp_ treatment compared to those following EMSC_Diss_ treatment, we next evaluated the quality of wound healing and the potential mechanisms behind wound healing. We harvested tissues from the wound area 14 days after treatment and confirmed expedited wound closure based on the presence of regenerated epidermis and dermal ridges. Specifically, epithelial “tongues” formed by keratinocytes migrating from the edge of the wound completely met in the center of the wounds in the EMSC_Sp_ group but not in the EMSC_Diss_ and vehicle groups (Fig. S3Aa). Much higher corresponding histological scores calculated based on the criteria listed in Table 1 24 were assigned to the EMSC_Sp_ group than the EMSC_Diss_ and vehicle groups (Fig. S3B).

**Figure 3:**
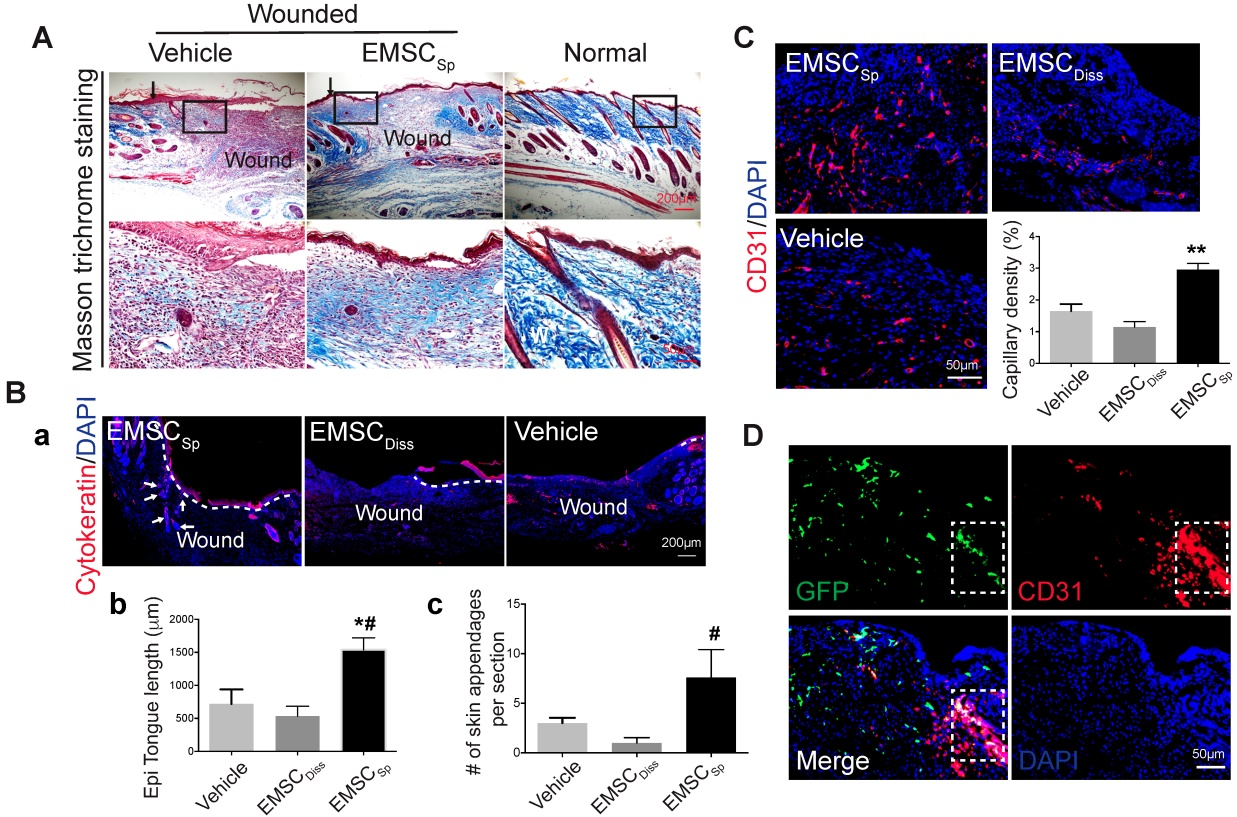
** Histological analyses of wounds with transplanted EMSC_Sp_.** (A) Masson trichrome staining of normal skin and wounds 14 days after treatment with EMSC_Sp_ or vehicle control. Boxed areas are amplified and shown on the bottom row. Arrows in the upper row indicate the junction between the wound and surrounding skin. (B) Re-epithelialization of wounds 14 days after treatment with EMSC_Sp_, EMSC_Diss_ or vehicle. Tissues isolated from the wounds were sectioned and immunostained (a) with anti-pancytokeratin antibody. White dashed lines indicate the detected epidermal keratinocytes, and white arrows indicate skin appendages. The epithelial tongue length (b) and the number of skin appendages per section (c) are shown in the bar charts. n = 5 biological repeats; *P < 0.05 for EMSC_Sp_ versus vehicle and ^#^*P* < 0.05 for EMSC_Sp_ versus EMSC_Diss_ per ANOVA followed by Tukey's test. (C) Detection of vascular endothelial cells in wounds 14 days after treatment as described above. Tissues isolated from the wounds were sectioned and immunostained with antibodies against CD31 as a marker for vascular endothelial cells. The results are quantified in a bar graph and displayed as capillary density. n = 5 biological repeats; **P < 0.01 for EMSC_Sp_ versus EMSC_Diss_ or vehicle per Kruskal-Wallis test. (D) Differentiation of transplanted EMSC_Sp_ into blood vessel endothelial cells. Wounds 14 days after EMSC_Sp_ (Envy) transplantation were isolated as described above and immunostained for both GFP (green) and CD31 (red), a marker for blood vessel endothelial cells. The white boxed area indicates double-positive cells.

During skin wound repair, local fibroblasts secrete extracellular matrix proteins, including collagen and fibronectin, that constitute newly formed granulation tissue, which induces the recovery of the structural integrity of the wounded area 25. Masson trichrome staining demonstrated more collagen deposition in wounds treated with EMSC_Sp_ than in those treated with vehicle 14 days after wound formation (Fig. 3A), indicating that EMSC_Sp_ increased granulation. In addition, more proliferative (Ki67^+^) cells were observed in the wounds treated with EMSC_Sp_ than in those treated with vehicle and EMSC_Diss_ (Fig. S3Ab and S3C). Furthermore, cells isolated from the EMSC_Sp_-treated wounds that were replated in a 6-well plate proliferated much faster than those isolated from the vehicle-treated wounds, as tracked in the IncuCyte Cell Monitoring system (Fig. S3D).

Immunostaining of the wounds after 14 days demonstrated that cytokeratin-positive cells in EMSC_Sp_-treated wounds carried out re-epithelialization much faster than those from mice in the vehicle- and EMSC_Diss_-treated control wounds (Fig. 3Ba and 3Bb). As quantified in Fig. 3Bc, more skin appendages, including hair follicles, were formed in the EMSC_Sp_-treated wounds than in the control wounds. Consistent with this, the hair follicle stem cell marker K15 was expressed in the wounds of the EMSC_Sp_ group but rarely observed in the wounds of the EMSC_Disss_ group (Fig. S3Ac). Furthermore, the number of microvessels (marked by CD31 in endothelial cells and αSMA in smooth muscle cells) was remarkably increased in the EMSC_Sp_-treated wounds compared with those in the vehicle- and EMSC_Diss_-treated control wounds (Fig. 3C, S3Ad, and S3E). Some CD31^+^ cells in the EMSC_Sp_-treated wounds were also GFP^+^ (Fig. 3D), suggesting that the transplanted EMSC were able to differentiate into vascular cells.

To determine whether EMSC treatment causes inflammatory cell aggregation, we tested granulocytes, including neutrophils, basophils, and eosinophils, for the pan marker LyG6. No obvious differences in the distribution and density of LyG6^+^ cells in wounds treated with or without EMSC_Sp_ or EMSC_Diss_ were observed (Fig. S3Ae and S3F), indicating that EMSC did not affect granulocyte aggregation in the wounds. We also analyzed the effect of EMSC on macrophage accumulation and polarization in wounds in the experiments described below.

### Transcriptomic analysis of transplanted and host cells in wounds

**Figure 4:**
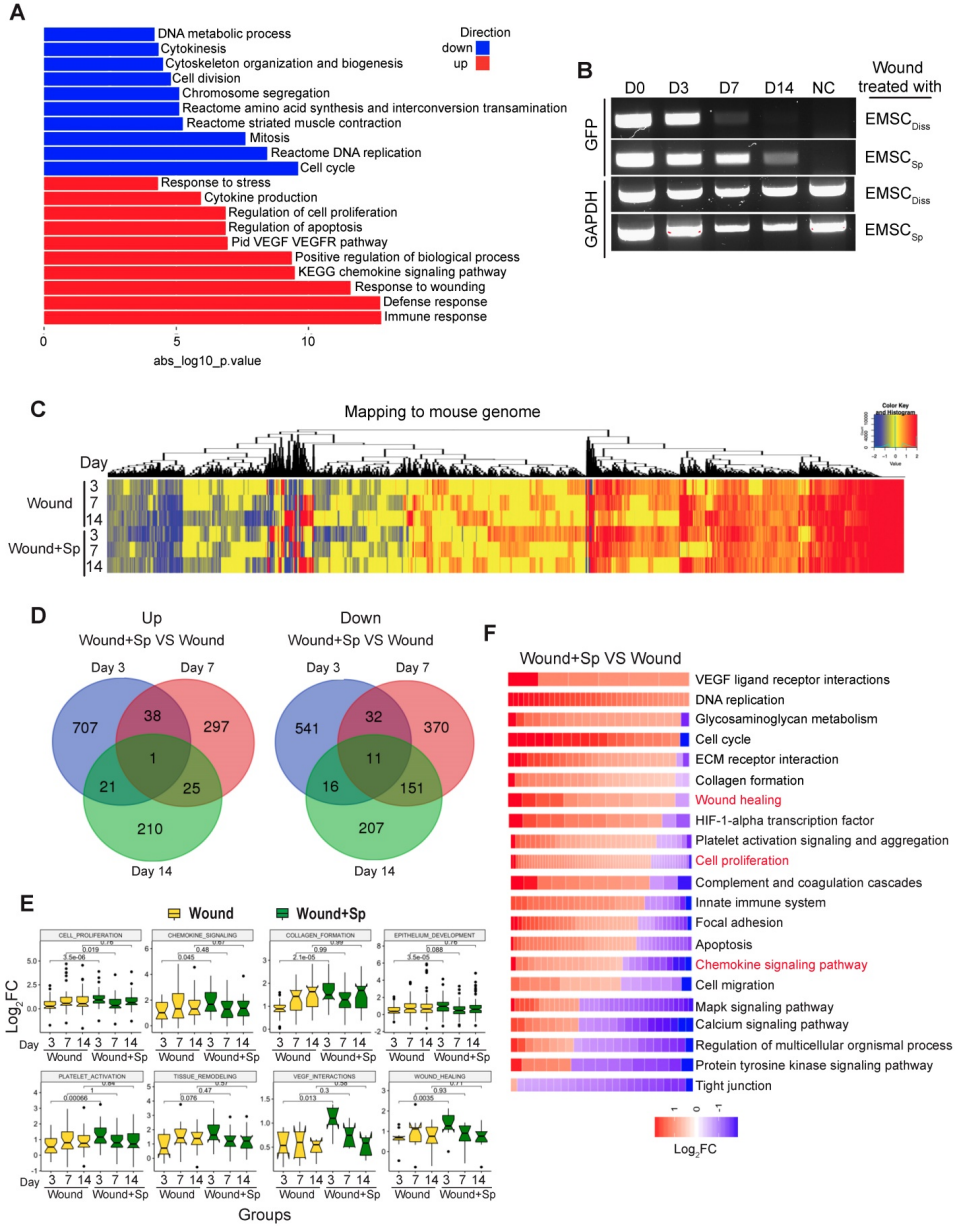
** Transcriptomic analysis of host cells in EMSC_Sp_-treated wounds.** (A) RNA sequencing of EMSC_Sp_ and EMSC_Diss_ before transplantation. Enrichment analysis of human DEGs in EMSC_Sp_ versus EMSC_Diss_. The bar length represents the -log10 *P* value. The red and blue bars represent terms enriched for up- and downregulated genes, respectively. (B) Detection of GFP mRNA in the wounds of NOD/SCID mice treated with GFP^+^ Envy EMSC_Sp_ or EMSC_Diss._ GFP transcript levels were detected via RT-PCR. GFP expression in H9 hESC-derived EMSC was used as a negative control. A GAPDH transcript with a conserved sequence between mice and humans was used as a loading control. (C) Heatmap displaying the transcriptomic changes following the treatment of wounds with EMSC_Sp_ (Wound+Sp) and vehicle (Wound) after various time points (3, 7, and 14 days). Expression levels of up- and downregulated genes that differed by more than 1.5-fold are highlighted in red and blue, respectively, as indicated in the color key above. (D) Venn diagram showing the intersections among up- and downregulated genes between the Wound+Sp with Wound samples at the three time points, as described above. (E) Boxplots showing changes in the expression of genes associated with eight wound healing-related pathways in the Wound (yellow) and Wound+Sp (green) samples at the three time points. Each plot stands for a pathway. (F) Heatmaps displaying significantly enriched DEGs categorized based on gene ontology. The expression level of up- and downregulated genes that differed by more than 1.5-fold changes are shown in red and blue, respectively, as indicated in the color key below.

To elucidate the molecular mechanism by which EMSC achieve their therapeutic effects by interacting with host cells, we conducted RNA sequencing (RNA-Seq) of skin wounds treated with EMSC_Sp_ or vehicle control. First, we detected the total mRNA in pre-transplantation EMSC_Diss_ and EMSC_Sp_ as controls and found that genes related to cell viability were dramatically increased while genes related to cell metabolism and proliferation were decreased in EMSC_Sp_ compared to EMSC_Diss_ (Fig. 4A) under ambient conditions 15. This suggests that EMSC_Sp_ possess unique features that may promote their therapeutic effects in wound repair. Second, since EMSC disappeared substantially faster from EMSC_Diss_-treated wounds than from EMSC_Sp_-treated wounds, as shown by immunostaining, flow cytometry (Fig. 2) and RT-PCR for GFP mRNA expressed in the Envy EMSC (Fig. 4B), we included wounds treated with vehicle, rather than those treated with EMSC_Diss_, as negative controls in the following transcriptomic analysis. EMSC_Sp_-treated wounds (Wound+Sp) and vehicle-treated control wounds (Wound) were dissected 3, 7, and 14 days after wound formation and immediately before transplantation as a normalization control for mouse gene expression. Some EMSC_Sp_ were also harvested right before transplantation (Sp) to serve as a normalization control for human gene expression.

We then mapped the sequenced reads to human and mouse genomes separately (Fig. S4A) and discarded the reads that mapped to the genomes of both species 26. By mapping the RNA-seq data to the mouse genome, we first identified a large number of differentially expressed genes (DEGs) with more than a 1.5-fold difference in their expression levels, including up- (UP) and downregulated (DN) genes (Fig. 4C). Based on Gene Ontology analysis, the UP genes in all 6 samples (versus those in day 0 Wound samples) were mostly related to wound healing and tissue regeneration, and the DN genes were mostly related to lipid biosynthesis and coenzyme metabolism, suggesting that wound healing occurred in both the treated and untreated groups. Furthermore, very few UP or DN genes overlapped among the three-time points, as shown in a Venn diagram (Fig. 4D), which was indicative of various biological processes involved at different healing stages.

Interestingly, many of the UP genes in the Wound+Sp group (versus the Wound group) on day 3 are related to biological processes involved in wound healing. However, the expression levels of these genes on days 7 and 14 decreased to levels similar to those in the corresponding controls (Fig. 4E). These expression patterns indicate that EMSC_Sp_ accelerates wound healing as early as 3 days after wound formation. We further analyzed gene ontology and signaling pathways in the samples collected after 3 days and found that specific gene groups were enriched in the UP and DN genes in the Wound+Sp group (versus the Wound group) on day 3 (Fig. 4F). Among them, the expression profiles of inflammation-related genes were analyzed (Fig. S4B). In the wounds that did not receive EMSC treatment, the expression of these genes increased and peaked on day 7 compared to their expression on day 0 and then slightly decreased on day 14. However, no such peak in expression was observed in the EMSC_Sp_-treated wounds. These profiles suggest that EMSC_Sp_ reduced inflammation in the wounded skin, which might have contributed to the accelerated healing observed in this group.

Next, we mapped the RNA-seq data to the human genome to identify changes in the gene expression profiles of EMSC_Sp_ before and after transplantation (Fig. S4C). We identified many UP genes and DN genes in EMSC_Sp_ 3, 7, and 14 days after wound formation, as shown in a heatmap (Fig. S4C). The expression of genes related to the acute inflammatory response declined, while the expression of genes related to cell proliferation and migration, chemokine signaling pathways, endothelial cell differentiation, the response to wounds, angiogenesis, and keratinocyte differentiation increased in the cells following transplantation (Fig. S4D and S4E).

### Potential CXCL12/Cxcr4 crosstalk between EMSC_Sp_ and host cells

Based on the mouse and human gene expression profiles in cells isolated from wounds treated with EMSC_Sp_ or vehicle, we assumed that the two profiles reflect the interactions between the transplanted cells and host cells. MSC exert therapeutic effects via cell empowerment and replacement, and transplanted MSC secrete many cytokines and growth factors to promote tissue repair 9. Among the human DEGs, those encoding members of the MSC secretome 27-29 (*IGF1*, *HGF*, *PDGFB* and *CXCL12*) were expressed at higher levels following transplantation than before (Fig. S4F), which was confirmed via immunostaining (Fig. S4G). To identify potential interactions between receptors and ligands (receptors expressed in mouse wound cells and ligands originating from the EMSC) during the wound healing response, we performed Pearson's correlation analysis. The expression of human *CXCL12* was highly correlated with the upregulated levels of mouse receptors (Fig. S4H). Since the chemokine signaling pathway was upregulated at the early stage of mouse wound healing (Fig. 4E and 4F) and CXCL12 plays a key role in the chemoattraction of endogenous cells to wounds 30-32, we decided to identify mouse genes triggered by human CXCL12.

**Figure 5:**
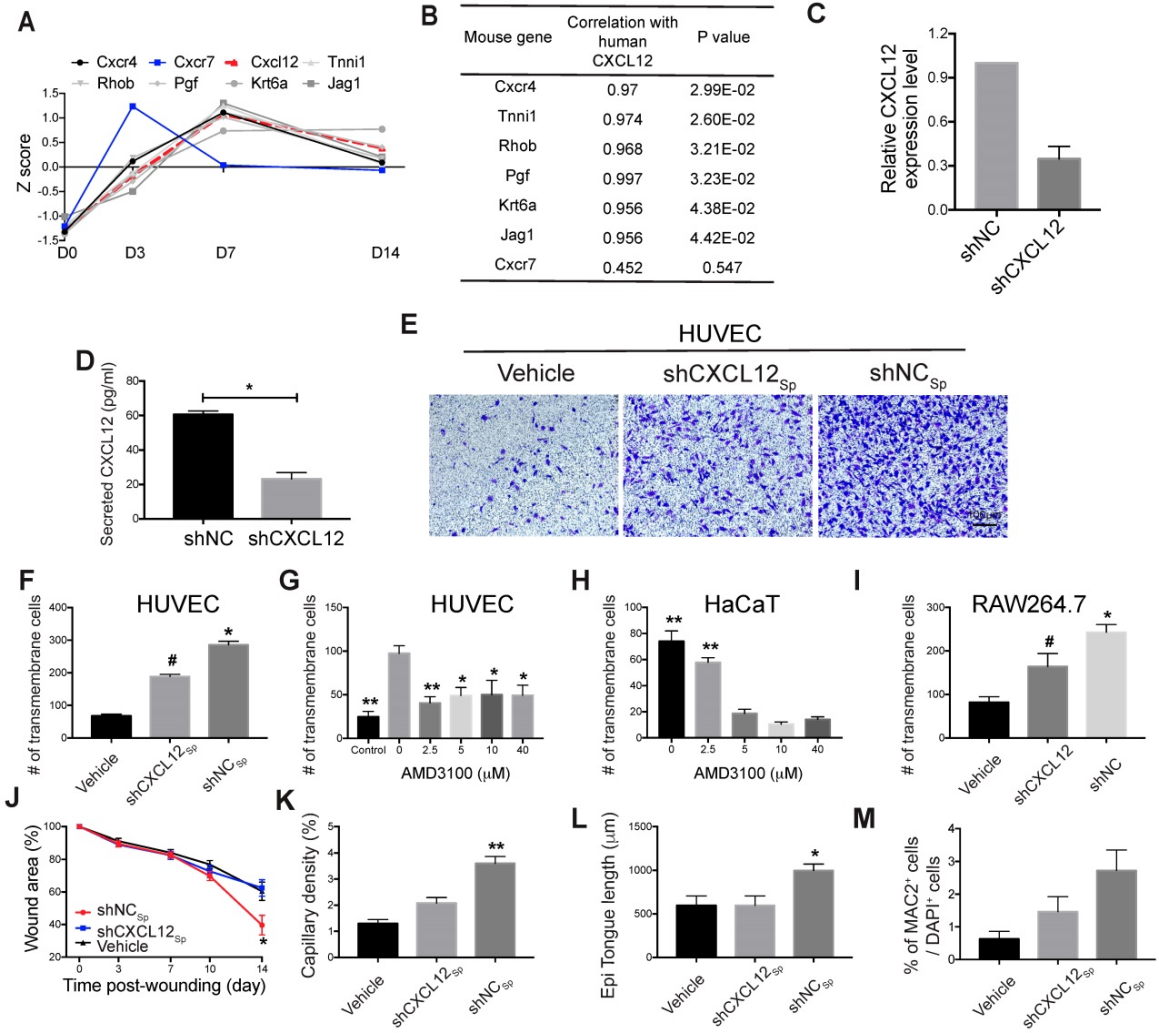
** Role of the CXCL12/Cxcr4 axis in EMSC_Sp_-promoted wound healing.** (A-B) Pearson's correlation coefficient between the expression of *CXCL12* in EMSC_Sp_ and the expression of Cxcl12-targeted genes in mouse cells based on RNA-seq data from wounds treated with EMSC_Sp_ and vehicle 0, 3, 7, and 14 days after wound formation (A). The X-axis represents the time, and the Y-axis represents the z-score values of the coefficient. The correlations between Cxcl12-targeted genes with *CXCL12* expression and their corresponding *P* values are listed in (B). (C) CXCL12 expression was decreased in EMSC stably transduced with lentivirus expressing shCXCL12 compared to that in EMSC transduced with lentivirus expressing shLacZ. The knockdown efficiency was determined by qPCR with 3 biological repeats. (D) CXCL12 secretion by EMSC transduced with shNC (control) or shCXCL12 was measured via ELISA. *P < 0.05 per the Mann-Whitney U test. (E-F) The migration of HUVECs towards EMSC_Sp_ expressing shCXCL12 was reduced compared to HUVEC migration towards EMSC_Sp_ expressing shNC in a Transwell assay. HUVECs were seeded on the top of the membrane insert, and no cells (vehicle, left), EMSC_Sp_ expressing shNC (shNC_Sp_, right) or shCXCL12 (shCXCL12_Sp_, middle) were seeded on the bottom of the Transwell insert. HUVECs that migrated across the membrane were stained with crystal violet and photographed under a microscope. The numbers of migrated HUVECs in the three groups are displayed in a bar chart and represent 3 biological repeats. *P < 0.05 for shNC_Sp_ versus shCXCL12_Sp_ or vehicle; and ^#^*P* < 0.05 for shCXCL12_Sp_ versus vehicle per ANOVA followed by Tukey's test. (G) Transwell assay to detect the EMSC_Sp_-oriented migration of HUVECs pretreated with AMD3100 (a CXCR4 antagonist) at various concentrations for 30 min. EMSC_Sp_ were seeded on the bottom of the Transwell insert to induce HUVEC migration. After incubation for 24 h, the number of HUVECs that migrated across the membrane was counted. The results were determined in three independent experiments. *P < 0.05 and **P < 0.01 versus 0 μM AMD3100 per ANOVA followed by Tukey's test. (H) Transwell assay to detect the EMSC_Sp_-oriented migration of HaCaT cells pretreated with AMD3100, with the number of cells that crossed the membrane counted as described above. The results were determined in three independent experiments. **P < 0.01 versus 5, 10 or 40 μM AMD3100 per ANOVA followed by Tukey's test. (I) Transwell assay to detect the EMSC_Sp_-oriented migration of RAW264.7 macrophages. The number of cells that crossed the membrane was counted as described above. The results were determined in three independent experiments. *P < 0.05 for shNC versus shCXCL12 or vehicle and ^#^*P* < 0.05 for shCXCL12 versus vehicle per ANOVA followed by Tukey's test. (J-M) Measurement of the wound area (J), capillary density (number of CD31^+^ cells among the total cells per wound section) (K), epithelial tongue length (L), and macrophage (MAC2^+^) percentage (M) in mice after transplantation with shNC_Sp_, shCXCL12_Sp_ or vehicle control. *P < 0.05 and **P < 0.01 for shNC_Sp_ versus shCXCL12_Sp_ or vehicle per one-way ANOVA followed by Tukey's or Dunn's posttest.

By calculating Pearson's correlation coefficients between human *CXCL12* and upregulated mouse genes, we found that the expression of murine *Cxcr4*, *Tnni1*, *Rhob*, *Pgf*, *Krt6a*, and *Jag1*, which are all *CXCL12* signaling effectors 33, was significantly correlated with human *CXCL12* expression (Fig. 5A and 5B, Table S1). These genes play important roles in wound healing, including the inflammatory response, cell migration, and angiogenesis 31, 34-38. In particular, *Cxcr4* encodes a well-known cognate receptor for Cxcl12, and the Cxcl12/Cxcr4 axis mediates MSC homing to sites of injury 30. Cxcr4 also participates in stem cell trafficking and vascular growth 39-41. Thus, our results indicate that human EMSC-derived CXCL12, which is highly homologous to and functionally indistinguishable from murine Cxcl12 42, might also bind murine Cxcr4 to enhance signaling, activate target genes in mouse cells, and recruit these cells to participate in wound healing. In contrast, we did not observe the differential expression of *Cxcr7* (another receptor for CXCL12) (Fig. 5A and 5B) or its related genes in wounds treated with or without EMSC_Sp_ (data not shown).

### Wound healing was impaired following inhibition of the CXCL12-Cxcr4 axis

To test the hypothesis above, we blocked the CXCL12/Cxcr4 axis to elucidate its role in EMSC-enhanced wound healing. We first knocked down CXCL12 in cultured EMSC by transducing the cells with a lentiviral vector expressing shRNA against CXCL12 (shCXCL12) or LacZ β-galactosidase (shNC) as a negative control (Fig. 5C). The level of CXCL12 protein secreted by shCXCL12-expressing EMSC was lower than that secreted by shNC-expressing EMSC, as determined via ELISA (Fig. 5D). Spheres composed of shCXCL12-expressing EMSC (shCXCL12_Sp_) had a slightly looser surface than spheres composed of shNC-expressing EMSC (shNC_Sp_). (Fig. S5A), and the proliferation of shCXCL12_Sp_ was slightly slower (but the difference was not statistically significant) than that of shNC_Sp_ (Fig. S5B). However, no marked difference in cell viability (Fig. S5C) and engraftment (Fig. S5G and S5Fc) was observed between the shCXCL12_Sp_ and shNC_Sp_ groups 14 days after wound formation. Notably, lentiviral shRNA transduction increased the secretion of the inflammatory cytokines IL-6 and IL-8 by cells transduced with either shCXCL12 or shNC compared to that in the non-transduced control cells (Fig. S5D).

Next, we compared the functions of shCXCL12_Sp_ and shNC_Sp_ *in vitro* and *in vivo*. Through Transwell assays, we found that shNC_Sp_ induced substantially more transmembrane migration of human umbilical vascular endothelial cells (HUVECs) than shCXCL12_Sp_ (Fig. 5E and 5F). Moreover, pretreatment with AMD3100 (a chemical inhibitor of CXCR4) dose-dependently inhibited the transmembrane migration of HUVECs (Fig. 5G) and human HaCaT keratinocytes (Fig. 5H). Consistent with this, *in vivo* studies showed that wound healing in mice was no longer expedited by shCXCL12_Sp_ following the topical application of the cell spheres (Fig. 5J and S5E). Less vascularization (Fig. 5K and S5Fa) and re-epithelialization (Fig. 5L and S5Fb) was observed in wounds treated with shCXCL12_Sp_ than in wounds treated with shNC_Sp_.

Macrophages play a variety of roles essential for both the early and late stages of wound healing, including roles in host defense, the promotion and resolution of inflammation, the removal of apoptotic cells, the support of cell proliferation, and tissue restoration 43, 44. To determine whether EMSC interact with macrophages in our wound model, we examined macrophage accumulation in the wounds and found that more macrophages (MAC2^+^ cells) were present in wounds treated with EMSC_Sp_ than in those treated with the vehicle control and that CXCL12 knockdown reduced macrophage recruitment (Fig. S5Fd and 5M). Consistent with this, a Transwell assay also showed much less migration of RAW 264.7 macrophages towards shCXCL12-transduced cells than shNC-transduced cells (Fig. 5I).

To further investigate the effects of EMSC on macrophages, we cultured EMSC and RAW 264.7 macrophages separately in a Transwell chamber. EMSC induced the remarkable polarization of the macrophage (Fig. S6A-C). Following co-culture, macrophages demonstrated higher levels of phagocytosis, as reflected by the ingestion of more *E. coli* than those ingested by control macrophages without preculture together with EMSC (Fig. S6D). These results suggest that EMSC can induce macrophage polarization and phagocytosis, which repress inflammation and promote wound repair, as previously reported 45.

Next, we verified *CXCR4*/*Cxcr4* expression in the three cell lines used in the Transwell assays. As shown in Fig. S5H and S5I, both HUVECs and HaCaT cells expressed *CXCR4,* and RAW264.7 cells expressed mouse *Cxcr4*, which is consistent with the literature 31, 46. Thus, all the results above suggest that CXCL12 produced by EMSC plays a crucial role in EMSC_Sp_-enhanced wound healing possibly by recruiting Cxcr4-expressing cells, such as vascular endothelial cells, keratinocytes, and macrophages.

### Biosafety evaluation of EMSC_Sp_ transplantation

**Figure 6:**
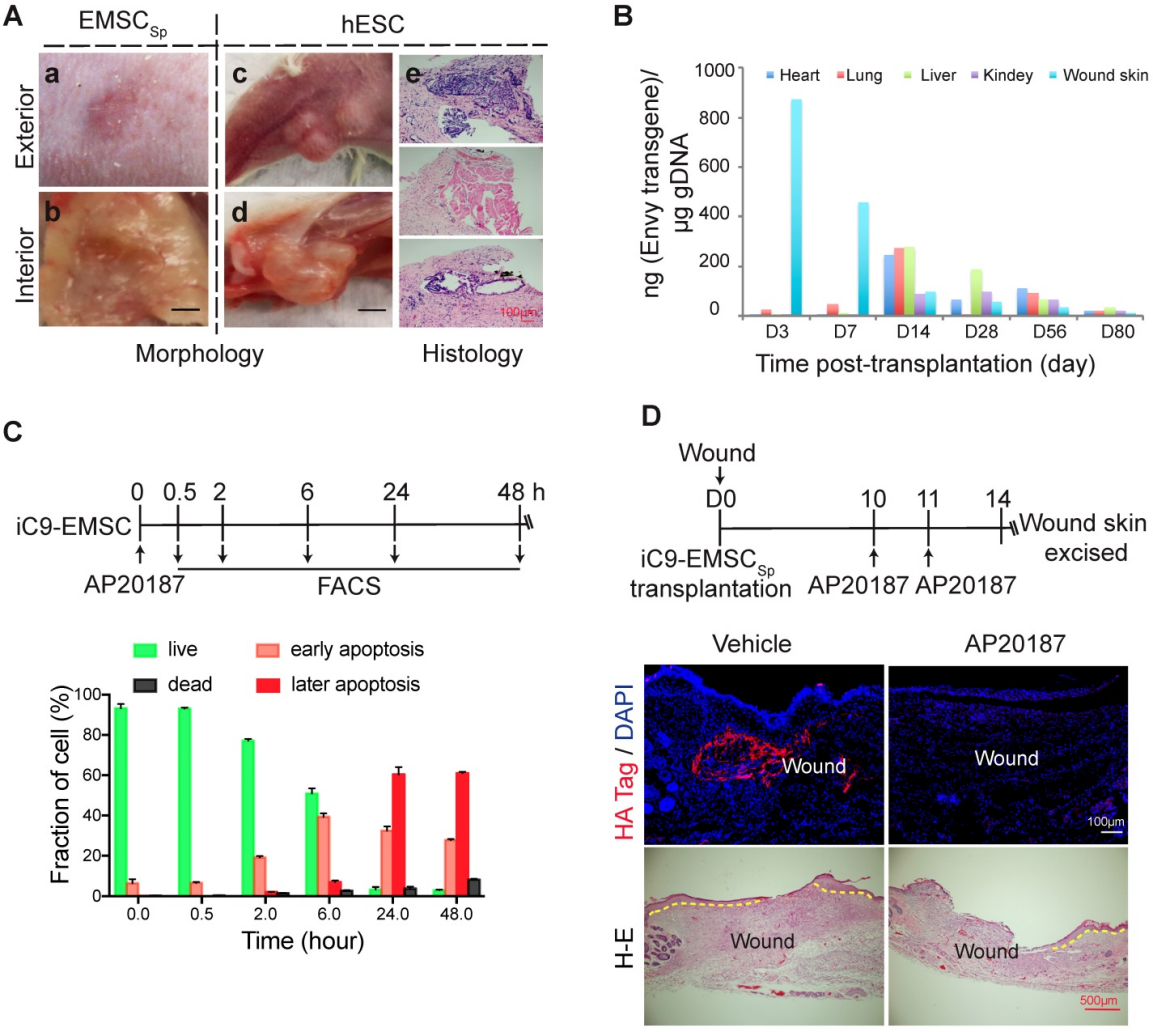
** Evaluation of the biosafety of EMSC_Sp_ transplantation and a strategy to establish safety-enhanced EMSC.** (A) Teratoma formation assay. Fifty-six days after wound formation, no tumors in the wound area transplanted with EMSC_Sp_ were observed based on photographs of the exterior (a) and interior (b) of the healed skin. In contrast, teratomas in the hind leg s.c. injected with hESC were observed in photographs of the exterior (c) and interior (d) of the injection site. Representative tissues from the three germ layers in sections of the teratomas were observed (e). (B) Quantitation of EMSC retained in mouse organs at various times following the transplantation with EMSC_Sp_ (Envy) onto wounds. The wounded skin, heart, liver, lung, and kidney were harvested at these time points and processed to isolate gDNA. The amount of GFP (ng) per μg of gDNA was used to determine the number of retained EMSC in each sample. (C) Apoptosis of iC9-expressing EMSC induced by AP20187 *in vitro*. As shown in the diagram above, after exposure to AP20187, the cells were harvested at the indicated times and processed for flow cytometry via AO/PI staining to determine the ratios of early and late apoptotic cells as well as dead cells. The results of three independent experiments are displayed as the mean ± SEM in a bar chart. (D) AP20187-induced elimination of iC9-EMSC from mice with iC9-EMSC_Sp_ transplanted onto the skin wound. As shown in the diagram above, AP20187 was i.p. injected into the mice at 10 and 11 days after wound formation, and the wound skin was dissected at day 14 and subjected to immunostaining for the HA Tag fused with the *iC9* gene transduced into EMSC. The samples were counterstained with DAPI to detect cell nuclei. HA^+^ cells were detected in samples from only mice without AP20187 treatment but not in those treated with the AP20187. The results were determined in four independent experiments. H-E staining in the bottom panel shows the extent of the re-epithelialization of iC9-EMSC_Sp_-transplanted wounds following treatment with the vehicle or CID.

The teratoma-forming ability of hESC 47 and the tumor-tropic effect of MSC 48 have caused great concern over their biosafety when used for therapeutic applications, and EMSC are obviously more worrisome than somatically derived MSC. To address these concerns, we first tested whether EMSC would form teratomas in NOD/SCID mice with hESC as a positive control. No tumors were found in the wounds of five mice 56 days after the transplantation of EMSC_Sp_ (Fig. 6Aa-b); in contrast, obvious teratomas were observed *in situ* in animals and cells derived from the three germ layers found in tumors by histology 56 days following the subcutaneous (s.c.) injection of hESC into the hind legs of the mice (Fig. 6Ac-e). This is consistent with reports that no teratomas were observed in immunocompromised mice transplanted with neural progenitors, osteogenic cells, and cardiomyocytes, which are other cell lineages derived from hESC 49-51.

Next, we analyzed the retention of EMSC derived from GFP^+^ Envy hESC in recipient mice. No GFP^+^ cells were found in sections of the wound skin, heart, liver, lung, and kidney 56 days after EMSC_Sp_ transplantation (data not shown). We then screened for the transgene, including part of its promoter and the subsequent N-terminal sequence of GFP (Fig. S7A), in the wound skin as well as some other organs in the animals via quantitative PCR (qPCR) (Fig. 6B). Three days after transplantation, the transgene was detected in mostly the wound skin, with a small amount detected in the lungs. Then, the transgene levels decreased in the wound and increased in the lungs on day 7. Later, similar transgene levels were detected in the heart, lungs, and liver, and relatively lower levels were detected in the kidney and wound skin on day 14. Furthermore, the transgene level had dramatically decreased in all of these tissues at days 28 and 56 and was barely detectable by day 80 (Fig. 6B). The GFP DNA disappeared faster in the wound skin of Balb/c mice than that of NOD/SCID mice per PCR analysis (Fig. S7A), indicating that EMSC might be subjected to stronger immune rejection in the immunocompetent Balb/c mice than in the immunodeficient NOD/SCID mice. Four months after the transplantation of EMSC_Sp_ or EMSC_Diss_, human gDNA for *hTK1* was undetectable in tissues isolated from the skin at the wound site and several other organs (Fig. S7B). These results suggest that, like somatically derived MSC 48, 52, EMSC (even when delivered as spheres) are discharged quickly from the recipients, which leaves little concern for tumorigenesis.

To firmly eliminate tumorigenic concerns about EMSC, we transduced H9 hESC with a suicide gene, inducible caspase 9 (iC9) 53, using a lentiviral vector to establish a stable cell line (iC9-hESC) and further differentiated iC9-hESC into MSC (iC9-EMSC) using our previously reported protocol 13. Apoptotic signs such as cell shrinkage and detachment occurred in iC9-EMSC as early as 30 min (data not shown) after treatment with AP20187, a noncytotoxic chemical inducer of dimerization 54. At 24 h, over 90% of iC9-EMSC had become apoptotic, as determined by flow cytometry via staining with PI and Annexin V-FITC, whereas untreated iC9-EMSC and AP20187-treated wild-type (WT) H9 EMSC both retained a normal morphology (data not shown) and contained fewer than 8.0% apoptotic cells (Fig. 6C and S7C). The cytotoxicity of iC9-EMSC after their exposure to AP20187 was further confirmed by Cell Counting Kit-8 (CCK-8) assay (Fig. S7D).

To assess the iC9 suicide system *in vivo*, we transplanted spheres formed by iC9-EMSC (iC9-EMSC_Sp_) onto wound surfaces in mice. The animals were then injected with AP20187 10 and 11 days after wound formation, and the retained EMSCs were identified via immunostaining for a human influenza hemagglutinin (HA) tag expressed from the same vector containing the iC9 sequence (but independent of AP20187) at day 14 (Fig. 6D). Almost no HA^+^ cells were found in the wounds of the AP20187-treated group, whereas a high density of HA^+^ cells was detected in the wounds of the untreated control group. These results suggest that the iC9 suicide system worked efficiently against EMSC_Sp_ both *in vitro* and *in vivo*. However, the injection of AP20187 10 days after wound formation delayed the re-epithelization of the EMSC_Sp_-treated wounds (Fig. 6D), indicating that EMSC are still required for the re-epithelization and closure of the wound at the late stage of wound repair.

## Discussion

Traditionally, dissociated MSC are injected intradermally into or around the wound area to promote healing 55, 56. However, this administrative method has limited therapeutic potential due to poor cell survival, engraftment, and retention at the injection site 14. In contrast, stem cell spheroids exhibit improved therapeutic efficacy in several types of ischemia models due to enhanced cell survival and retention 57. In this study, for the first time, we employed human EMSC as an endless cell source to treat an excisional wound splinting mouse model 16 and topically applied EMSC_Sp_, *i.e.*, simply dropped the spheres onto the wound surface instead of their subcutaneous injection—a widely used method. We observed expedited wound healing accompanied by increased survival and infiltration of the transplanted cells into wounds treated with the new method compared with those following the application of EMSC_Diss_ with a traditional transplantation method, which surprisingly promoted even slower healing than the vehicle control.

The following reasons may explain the enhanced survival, infiltration, and retention observed in skin wounds treated with EMSC_Sp_ compared to those in skin wounds treated with EMSC_Diss_. First, tight cell-cell contact within EMSC_Sp_ can prevent anoikis and promote cell survival, as shown in our recent study 15. Second, the relatively hypoxic core of a stem cell spheroid, like that of an EMSC_Sp_, preconditions the cells to tolerate the ischemic and hypoxic conditions in the wound, which increases survival and engraftment compared to those observed following treatment with dissociated cells 58.

After their survival and engraftment, EMSC expedited wound healing, perhaps mainly at the early stage of wound healing, as shown by the RNA-seq data. Genes involved in the extracellular matrix (ECM) organization, DNA replication, glycosaminoglycan (GAG) metabolism, platelet activation signaling and aggregation, wound healing, and the chemokine signaling pathway were upregulated, whereas genes involved in inflammation were downregulated compared with their expression in the vehicle control-treated group. The increased collagen deposition and Ki67^+^ cell number in grafted wounds at later stages of wound healing suggest that EMSC also promote ECM reorganization and stabilization and cell proliferation following their initial effects on wound repair.

Moreover, the induction of neovascularization was more remarkable in EMSC_Sp_-treated wounds than in the corresponding controls. The formation of new blood vessels is necessary to sustain newly formed granulation tissue and maintain the survival of keratinocytes. Interestingly, a small number of vascular cells in Envy EMSC_Sp_-treated mice expressed GFP, indicating that wound healing may also benefit from the direct differentiation of EMSC into vascular cells.

To our surprise, the expedited wound repair following EMSC_Sp_ treatment was observed in not only immunocompromised but also immunocompetent mice, suggesting that the murine immune system does not compromise the efficacy of human EMSC or that their efficacy does not depend on the immunomodulatory effects of EMSC, as demonstrated in other disease models 12, 13, 15. We also did not observe a remarkable difference in the accumulation of granulocytes in wounds treated with or without EMSC_Sp_. However, it would be intriguing to elucidate how EMSC interact with specific immune cells, including T cells, B cells, and natural killer cells, in the host and whether these interactions contribute to wound healing in immunocompetent mice.

Next, we sought molecular evidence of the mechanism by which EMSC_Sp_ affects host cells during wound healing by focusing on the CXCL12-Cxcr4 axis. We previously found that *CXCL12* expression is upregulated in EMSC cultured as 3D spheroids as opposed to in a monolayer 15. Here, the expression of *CXCL12* in EMSC_Sp_ (Fig. S4G) and Cxcl12 target genes in the host cells (Fig. 5A) was higher after EMSC_Sp_ transplantation to the wound than before transplantation, which may have been further induced by the ischemic and hypoxic conditions in the wound center. Increasing evidence has demonstrated the pivotal role of CXCR4 in the biological functions of MSC 59, 60 and MSC-mediated tissue repair and regeneration in the liver, heart, kidney, and skin 61-65. Genetically engineered MSC expressing ectopic CXCL12 were shown to enhance wound healing 66. Lentiviral transduction not only specifically reduced *CXCL12* expression but also nonspecifically increased the expression of the inflammatory cytokines IL6 and IL8, although there was no significant difference in the cytokine levels between shNC- and shCXCL12-transduced cells. CXCL12 knockdown in EMSC abolished the accelerated wound healing and reduced vascularization, re-epithelialization, and macrophage accumulation in wounds, whereas inhibition of the CXCL12-CXCR4 axis reduced the migration of CXCR4^+^ cells (human vascular cells, epithelial keratinocytes and macrophages) towards EMSC_Sp_ *in vitro*. Thus, the CXCL12-Cxcr4 axis may play an essential role in recruiting the murine counterparts of these Cxcr4^+^ cells and retaining CXCR4^+^ EMSCs themselves in the wound to promote wound repair.

Finally, we addressed the biosafety of EMSC_Sp_ (Fig. 6 and S7) based on concerns regarding the tumorigenicity of hESC and the tumor-tropic effect of MSC. First, teratomas were observed in only NOD/SCID mice s.c. injected with hESC but not EMSC. Second, following the topical application of EMSC_Sp_ to skin wounds, levels of the transgene contained in the EMSC in the treated wound declined dramatically within 14 days; transgene levels increased in some tested organs (the heart, lungs, liver, and, to a lesser level, the kidney) after 14 days but then decreased gradually and were barely detectable at day 80. Four months after transplantation, no human gDNA was detected, and no tumor was found in the wound skin or several other organs of the EMSC_Sp_-treated mice. This is consistent with the rapid disappearance of exogenous MSC delivered to animals via other methods 48, suggesting that long-term engraftment is not necessary for the therapeutic effects of EMSC in the mouse model. By using iC9-EMSC, we were able to kill most of the EMSC *in vitro* 24 h following treatment with the AP20187 and remove almost all the engrafted cells in EMSC_Sp_-treated wounds five days after the intraperitoneal (i.p.) injection of AP20187 into the animals. Thus, the rapid clearance of EMSC from the recipient and the use of the suicide-inducible iC9 system both ensure the biosafety of EMSC_Sp_ in clinical applications.

**Figure 7:**
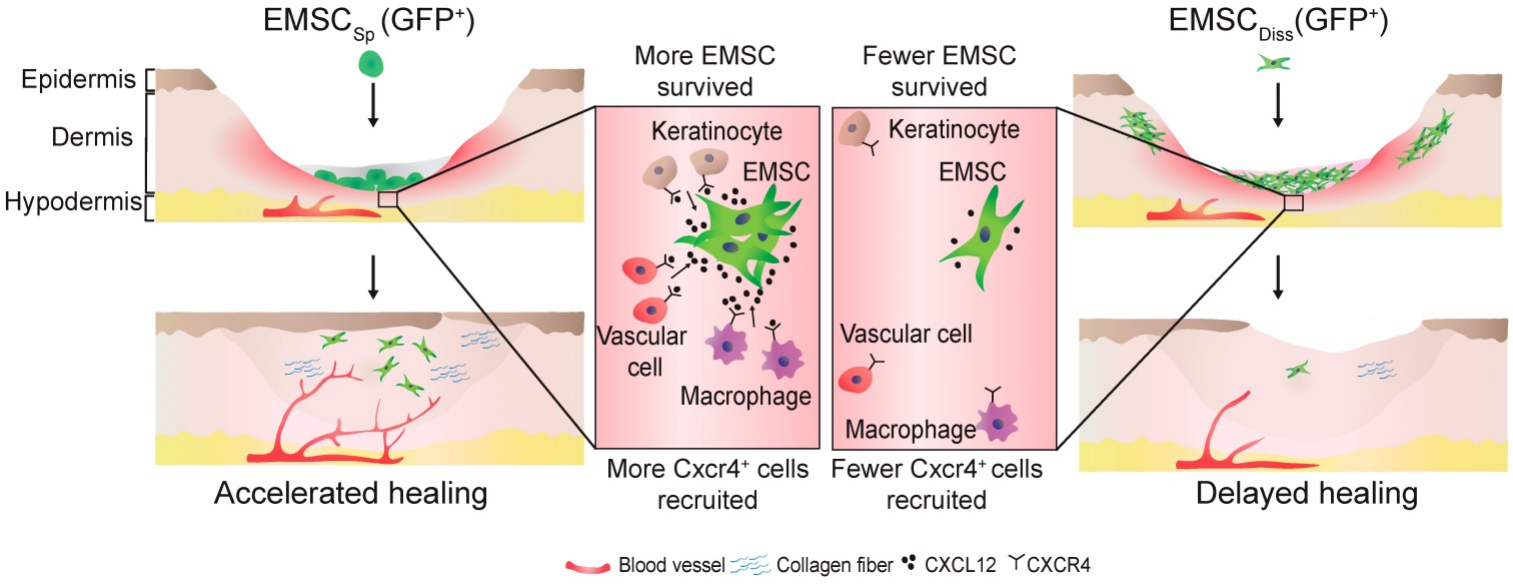
Schematic to summarize the findings of this study.

In conclusion, this study demonstrates the beneficial effect of EMSC_Sp_, which outperformed EMSC_Diss_, in cutaneous regeneration and wound healing in immunodeficient and immunocompetent mice. As summarized in Fig. 7, cell empowerment played a major role in the expedition of wound repair, which was reflected by the enhanced proliferation of endogenous cells, neovascularization, and re-epithelialization, whereas cell replacement also participated in healing, as evidenced by the direct differentiation of EMSC into vascular cells. Signaling between EMSC and host cells through the CXCL12-Cxcr4 axis is required for efficacious treatment. More importantly, long-term engraftment of EMSC is not required for their efficacy, and the engrafted cells can be cleared either naturally or via the administration of a chemical inducer. Thus, our study presents the noninvasive application of EMSC_Sp_ as a novel, convenient, efficacious, and safe method for the treatment of skin injury.

## Materials and Methods

### MSC preparation and spheroidal formation

All the methods were carried out in accordance with the National Institutes of Health Guidelines on Human Stem Cell Research. All the experimental protocols were approved by the University of Macau Sub-panel of Biomedical Science and Engineering Research Ethics. The Envy (GFP^+^) 18, CT3 20 and H9 21 hESC lines were used in this study. EMSC were generated by inducing the differentiation of hESC into MSC using our previously reported method 13. EMSC were identified by fluorescence-activated cell sorting (FACS) (Fig. S8). BM-MSC were derived from bone marrow aspirates donated from healthy male adults using a protocol approved by the University of Macau Panel of Research Ethics described before 12.

The resultant MSC (including EMSC and BM-MSC) were cultured in high-glucose Dulbecco's modified Eagle's medium supplemented with 20% fetal bovine serum at 37.0℃ in a 5% CO_2_ incubator and passaged every 5-7 days. MSC between passages 6-10 were used for all experiments. To form spheroids, MSC were split and seeded on adhesion substrates at a density of 2.5×10^4^ cells/drop by using the hanging drop method, as we recently reported 15, and spheroids formed in the hanging drop within 2 days of culture. Either MSC spheroids or cells dissociated from MSC in a monolayer were harvested for transplantation.

### Mouse excisional wound splinting model and EMSC transplantation

All the animal experiments were conducted under an animal use protocol approved by the University of Macau Sub-panel on Animal Research Ethics. NOD/SCID mice or Balb/c mice (6-8 weeks old; male; body weight, 20-23 g) were obtained from the animal facility of the University of Macau. An excisional wound splinting model was generated as reported 16. In brief, after the removal of hair from the dorsal surface and anesthetization of the mouse, a biopsy punch was used to make two full-thickness excisional wounds, each 5 mm in diameter, on each side of the midline. The animals were then randomly divided into three groups.

For the EMSC_Sp_ group, 40 spheres equivalent to 1×10^6^ EMSC, were directly dropped onto the surface of each wound immediately after its formation. For the EMSC_Diss_ group, 0.7×10^6^ EMSC_Diss_ in 100 μl PBS (70% of the total cell dose) per wound were evenly injected into the dermis in four spots around the wound, and 0.3×10^6^ EMSC_Diss_ (30% of the total cell dose) mixed with 30 μl Matrigel were dropped onto the surface of the wound. For the vehicle control group, 100 μl PBS alone per wound was evenly injected around the wound, and 30 μl Matrigel was dropped onto the surface of the wound as described above. A donut-shaped silicone sheet was placed on each wound to center the wound within the splint. An immediate-bonding adhesive was used to fix the splint to the skin, followed by interrupted sutures to stabilize its position, and the wounds were dressed first with transparent Tegaderm and then with self-adhering elastic bandages.

Photographs of the wound areas were taken 0, 3, 7, 10, and 14 days after wound formation. Wound closure refers to re-epithelialization of the wound bed indicated by the gradual extension of the wound margin towards the center of the wound and a reduction in the wound area. The wound margin was marked on the images, and the wound area was calculated with an image analysis program (www.getpaint.net). The investigators who measured the wounds were blinded to the treatment groups. The percentage of the wound area was calculated as follows: (area of current wound/area of the original wound) × 100%. Mice were sacrificed at the designated time points, and skin samples from the wound and 2 mm of the surrounding skin were harvested using an 8 mm biopsy punch. All results from the two wounds in each mouse were averaged.

To track iRFP-expressing EMSC transplanted into wounds in live mice, the animals were first anesthetized, and images were captured using the Bruker In-vivo Xtreme imaging system. A deeply cooled 4 MP CCD camera was used for photography with the following parameters: f-stop, 4; binning, 2×2; acquisition time, 1 sec; excitation filter, 630 nm; emission filter, 700 nm; and exposure time, 60 sec. Signal intensities were represented by radiance and encoded by pseudocolors on the iRFP images.

### Assays of EMSC isolated from EMSC-treated wounds

Following transplantation with EMSC_Sp_ (Envy), wound skin tissues were excised above, and dispersed into single-cell suspensions as previously described 67. In brief, the tissue was incubated with 0.5% Trypsin overnight at 4℃, minced completely, and incubated in a digestion buffer containing collagenase I (2.75 mg/ml), and DNase I (150 U/ml) in a 37℃ shaking bath for 2 h. The trypsin digests were pooled and filtered through a nylon cell strainer with the pore size at 40 μm. Cells were pelleted and re-suspended in PBS containing 3% FBS, and the number of GFP^+^ cells determined among 50,000 events via flow cytometry.

Some of the isolated cells were plated on a 96-well plate at the same initial density for each group, and the plate was placed in the IncuCyte ZOOM^TM^ system. Phase-contrast images were captured at 2 h intervals for a total duration of 90 h. Percentage of the confluence of each well was calculated using the IncuCyte ZOOM^TM^ software, which is proportional to the cell proliferative rate and comparable between cells at similar sizes.

### RNA-Seq

Wound skin tissues were harvested as above, and three wounds were pooled per group. The tissues were sliced into small pieces and homogenized with a pestle. Skin fragments were added into a lysis buffer provided by QIAGEN RNeasy kit to isolate total RNA according to the manufacturer's instructions. The integrity and quality of samples were verified using the Nano RNA Bioanalyzer Assay (Agilent Technologies). Samples with RNA integrity number larger than 9 were used for Next Generation Sequencing library preparation using the NEB Ultra Directional RNA Library Preparation Kit.

Pair-end sequencing was performed on the Illumina HiSeq2500 platform. Firstly, we applied FastQC for standard quality control of our 100-bp length, paired-end RNA-Seq data generated by Illumina HiSeq2500, tested both the raw sequence data, and trimmed sequence data. Trimmomatic was used to trim the adaptor, and discarded low-quality reads and the reads with length less than 36 bp. After trimming, we reserved the paired reads and abandoned unpaired reads for next step analysis. Then cleaned reads (paired reads) were submitted to the tuxedo pipeline. Tophat2 internally called bowtie2 to map raw reads against the human genome hg38 and mouse genome mm10 with 2 mismatches allowed. Finally, cufflinks were applied to generate the Fragments Per Kilobase Million (FPKM) value for each gene, based on the hg38 or mm10 gene annotation GTF file of University of California Santa Cruz. Human and mouse genome sequences, annotation files, and genome indexes were downloaded from iGenome.

The RNA-seq data was statistically analyzed with R. One-way ANOVA test and fold change were used to identify DEG. We downloaded all the gene sets used for function/pathway enrichment from Gene Set Enrichment Analysis of the Broad Institute, and elucidated the hypergeometric distribution to determine *P* values of each enriched pathway. The R package “gplots” and “ggpubr” were used to prepare all figures.

### Data analysis

Experimental data were analyzed using GraphPad Prism7. All values are expressed as mean ± SE. One-way ANOVA or Kruskal-Wallis test was used for multiple group comparisons, two-tailed Student's *t*-test or Mann-Whitney *U* test was performed for comparison between two groups. Statistical significance was defined as *P < 0.05 and ** *P* < 0.01. All flow cytometry data were analyzed and generated using FlowJo 7.6. All figures were prepared using GraphPad Prism.

## Supplementary Material

Supplementary materials and methods, figures and tables.

## Figures and Tables

**Table 1:** Criteria for histological score of skin wounds

Score	Re-epithelialization	Cell infiltration	Granulation	Angiogenesis
1-3	Minimal (0-40%)	Thin cell layer	Scant	< 10 vessels/HMF
4-7	Moderate (40-70%)	Moderate cell layer	Moderate	10-20 vessels/HMF
8-10	Complete (70-100%)	Thick cell layer	Abundant	> 20 vessels/HMF

HMF: High-magnification field

## Abbreviations

AO/PI: acridine orange & propidium iodide
BM-MSC_Sp_: MSC spheres derived from human bone marrow
CCK-8: cell counting kit-8
DEG: differentially expressed genes
EMSC: hESC-derived MSC
EMSC_Diss_: dissociated EMSC
EMSC_Sp_: EMSC spheres
Epi: epithelial
GFP: green fluorescent protein
HA: hemagglutinin
HaCaT_Sp_: HaCaT cell-formed spheres
H-E: hematoxylin and eosin staining
hESC: human embryonic stem cells
HUVEC: human umbilical vascular endothelial cells
iC9: inducible caspase-9
iC9-EMSC: iC9-expressing EMSC
iC9-EMSC_Sp_: iC9-EMSC-formed sphere
iRFP: infrared red fluorescent protein
M1: M1 macrophage
M2: M2 macrophage
MSC: mesenchymal stem cells
RAW: RAW264.7 macrophage
RNA-seq: RNA sequencing
shCXCL12: shRNA against CXCL12
shCXCL12_Sp_: sphere formed by shCXCL12-expressing EMSC
shNC: shRNA against the LacZ β-galactosidase as a negative control
shNC_Sp_: sphere formed by shNC-expressing EMSC
TK1: thymidine kinase 1
Wound+Sp: EMSC_Sp_-treated wounds
WT: wild-type
