# Supplementary material for: Noninvasive application of mesenchymal stem cell spheres derived from hESC accelerates wound healing in a CXCL12-CXCR4 axis-dependent manner
Source: Theranostics. 2019 Aug 14;9(21):6112–28. doi: 10.7150/thno.32982 (PMC6735514; doi:10.7150/thno.32982)
Supplement: Supplementary file 1 — Supplementary materials and methods, figures and tables. [file thnov09p6112s1.pdf]

Supplemental Information

Noninvasive application of mesenchymal stem cell spheres derived from hESC accelerates wound healing in a CXCL12-CXCR4 axis-dependent manner

Xiaoyan Wang, Bin Jiang, Huiyan Sun, Dejin Zheng, Zhenwu Zhang, Li Yan, Enqin Li, Yaojiong Wu, Ren-He Xu

Supplemental Materials and Methods

Vector construction

For the construction of the lentiviral vector pCDH-CAG-iRFP682-T2A-Puro, iRFP682 was amplified from piRFP-N3-Tyrosinase (a gift from Santiago Di Pietro via Addgene #80152) [1] and inserted into the NotI/EcoRI site of pCDH-CAG-MCS-T2A-Puro, which was modified from pCDH-EF1-MCS-T2A-Puro (Systembios). The resultant pCDH-CAG-iRFP682-T2A-Puro vector was packaged in 293T cells via co-transfection with the envelope vector pMD2.G (gift from Didier Trono, Addgene # 12259) and packaging vector pCMV delta R8.2 (a gift from Didier Trono via Addgene

#12263) using Lipofectamine 3000 reagent (Invitrogen). Lentiviruses were collected and used to transduce EMSC, followed by puromycin selection for 2 weeks.

For the construction of the lentiviral vector pCDH-CAG-iC9-P2A-Neo, iC9 gene was amplified from pMSCV-F-del Casp9.IRES.GFP (a gift from David Spencer via Addgene #15567) [2] and inserted into EcoRI/StuI sites of P2A-Neo to obtain iC9-P2A-Neo cassette, in which Neo was amplified from PC5Kan-P2A and inserted into BglII/PmeI sites of the same plasmid (a gift from Barry Ganetzky via Addgene #51814). iC9-P2A-Neo cassette was then cloned into EcoRI/SalI sites of pCDH-CAG-MCS-T2A-Puro as described above to obtain the pCDH-CAG-iC9-P2A-Neo. The resultant lentiviral vector was packaged as described above. Lentiviruses were collected to transduce H9 hESC, followed by G418 selection for 2 weeks. Single cell-derived colonies were picked via serial dilution to establish the iC9 stable hESC line.

For the construction of the shCXCL12 lentiviral vector, oligonucleotides for shCXCL12 were synthesized and annealed, and the resultant DNA fragment was cloned into the KpnI/EcoRI sites of pLKO_TRC005. The shLacZ lentiviral vector TRCN0000072225 (a gift from William Hahn via Addgene #78160) was used as a

control. Both vectors were packaged as described above, and the resultant lentiviruses were collected and used to transduce EMSC followed by puromycin selection for 2 weeks. Primers and oligonucleotides used for the vector constructions are listed in Table S2.

Histologic examinations and immunostaining

Tissue specimens were fixed in 4% paraformaldehyde, dehydrated in a graded ethanol series and embedded in paraffin. Sections in 5-μm thickness were stained with hematoxylin and eosin for microscopy. Each slide was given a histological score, according to parameters including re-epithelialization, cell infiltration, granulation formation, and angiogenesis as reported [3] with slight modification as detailed in Table 1. For Masson trichrome collagen staining, the whole procedure was performed according to the manual of Heart Biological Technology.

Immunofluorescence staining was performed using a standard procedure. In brief, after antigen retrieval, tissue sections were treated with 0.3% Triton X-100 for 10 min. and washed extensively in PBS, blocked with 5% BSA for 1 h, then the sections were

stained with specific primary antibodies: EMSC (Envy), endothelial cells, smooth muscle cells, keratinocytes, and macrophage were detected by antibodies against GFP, CD31, SMA, epidermal keratin subunits, and MAC2, respectively with the nucleus counterstained with DAPI. Cell proliferation was detected with a primary antibody against Ki67. All the staining was visualized with fluorescent-conjugated secondary antibodies. Images were captured with Carl Zeiss Axio Observer microscope.

Cell migration via Transwell assays

The chemotactic motility of cells was performed using a Transwell plate with an upper insert containing a polycarbonate membrane at 3.0-μm pore size (Corning). For migration assay, 2 x 10^5 HUVEC or macrophage per well were suspended in fresh medium and seeded into the upper insert, and spheres containing a total of $1x10^6$ EMSC were seeded in the lower compartment. After 24-h incubation, cells migrated across the membrane of the insert were stained with 0.5% crystal violet and counted in seven random fields using a light microscope at 20X magnification. For inhibition of CXCR4,

HUVEC or HaCaT cells were pretreated with AMD3100 for 30 min, followed by thorough washing and seeding the cells to the insert of Transwell as above.

Detection of EMSC retained in various organs of the host following transplantation

At various days after transplantation of $EMSC_{Sp}$ (Envy) onto mouse wounds, genomic DNA (gDNA) was isolated from the wound skin and some major organs of the mice, using a gDNA extraction kit (Tiangen), according to manufacturer's instructions. The DNA concentration and purity were estimated by measuring the optical density. Two hundred ng of purified DNA from each sample were amplified using the TaqMan Universal PCR Master Mix. Testing of target DNA fragments was performed via real-time quantitative PCR (qPCR) with primers and probes for the transgene and *GAPDH* listed in Table S2. gDNA isolated from 1×10^6 Envy hESC was used as a positive control, and a standard curve was generated using qPCR to calculate the amount of the transgene (ng) per μg of gDNA, which reflects the relative number of EMSC (Envy) retained in each sample.

For long-term observation, four months after transplantation with $EMSC_{Sp}$ or $EMSC_{Diss}$ onto wounds, gDNA from the wound-site skin and several other organs were isolated. qPCR was performed to measure the amount of gDNA for human thymidine kinase-1 (*hTK1*) [4]. Vehicle-treated control was tested as the negative control, and EMSC-transplanted day-1 wound as positive controls. The *hTK1* level was normalized by the amount of total gDNA.

Teratoma formation assay

1×10^6 hESC or EMSC were injected subcutaneously into a hind leg of NOD/SCID mice. After 8 weeks, resulting teratomas were surgically dissected out of the mice and fixed with 4% paraformaldehyde. The samples were embedded in paraffin, sectioned into 5-μm slices, and stained with hematoxylin and eosin.

Assays of apoptotic, live, and dead cells

For EMSC stably transduced with iC9, the AP20187 was added at 20 nM to the cell culture. The cells were harvested at various times, stained with Annexin V and propidium iodide (PI) to determine the percentage of apoptotic cells via flow cytometry.

For isolated skin wound, the tissues were digested as described above, and resultant single cells stained with acridine orange (AO)/PI. The stained cells were photographed and quantified on Cellometer.

Enzyme-linked immunosorbent assay (ELISA)

Condition medium was collected as above, CXCL12 protein was measured by using an ELISA kit (Thermo Scientific) according to the standard procedures provided by the manufacturer. A microplate reader was used to read the optical density at 450 nm.

EMSC and macrophage interaction assay

Co-culture of EMSC with RAW264.7 macrophage was performed in a Transwell plate with an inset per well which contained a polycarbonate membrane with an 8.0-μm pore size (Corning). Two x 10^5 EMSC per well were suspended in fresh medium and seeded in the upper insert, and 2 x 10^5 RAW macrophage seeded in the lower compartment. After 48-h incubation, the insert with EMSC was removed, and the macrophages in the bottom were subjected to the following experiments, either immunostaining for the M2

macrophage marker CD206 or qRT-PCR with primers whose sequences are listed in Table S2.

Macrophage phagocytosis assay

EMSC or nothing seeded in the upper insert of a Transwell and RAW264.7 macrophage seeded in the lower compartment were co-cultured as above. Forty-eight hours later, the macrophage were incubated with GFP-labeled E. coli bioparticles (200 μg/ml). After 1-h incubation, the cells were fixed, counterstained with DAPI for the nuclei, and photographed. The number of macrophage ($DAPI^+$) with phagocytosis (GFP^+) over the total number of macrophage per view was calculated as % of phagocytosis.

Supplemental Tables

Table S1. Murine genes with differential expression relevant to the expression of human *CXCL12* in mouse skin wounds transplanted with human $EMSC_{Sp}$*

Gene Ontology #	Function	*P* value	Differentially expressed gene in mouse cells
0006955	Immune response	5.63E-03	*Zgpat, Serpina3g, Cxcl2, Cd300e, Acp5, Tgtp1, Rnf19b, Il7r, Fth1, B2m*

0048729	Tissue morphogenesis	6.45E-03	*Aldh1a2, Krt6a, Pgf, Smad7, Ptch1, Jag1, Tnni1*
0001944	Vasculature development	8.13E-03	*Aldh1a2, Pgf, Smad7, Cxcr4, Dll4, Rhob, Vash1*
0008285	Negative regulation of cell proliferation	2.04E-02	*Trim35, Cd274, Ptch1, Pmaip1, Fth1, Vash1*
0006631	Fatty acid metabolic process	1.57E-09	*Acsm3, 9130409123rik, Ech1, Prkag1, Elovl3, Fa2h, Olah, Prkag2, Aacs, Acaa1a, Slc27a4*
0006732	Coenzyme metabolic process	2.15E-03	*Coq3, Pank1, Acly, Pdss1, Mgst1*
0055114	Oxidation reduction	3.75E-03	*Tm7sf2, Cyp51, 9130409123rik, Cyr1a1, Fa2h, Bckdhb, Decr1, Etfa, Nsdhl*

*The data are derived RNA-seq of mouse skin wounds at 3, 7, and 14 days after transplantation with human $EMSC_{Sp}$

Table S2. Primers and oligos for vector construction, and detection of gDNA and cDNA

**Primers and oligos**	**Sequence (5'-3')**	**Note**

iRFP-F	AAAAGAATTCGCCGCCACCATGGCGGAAGGATCCGTCG	To amplify *iRFP682* from a parental vector
iRFP-R	AAAAGCGGCCGCCTCTTCCATCACGCCGATCTGC	To amplify *iRFP682* from a parental vector
iC9-F	AAAAGAATTCGCCACCATGCTTGAGGGAGTGCAGGTGGAGAC	To amplify *iC9* from a parental vector
iC9-R	CCTGTCGAGTGCGTAGTCTGGTACG	To amplify *iC9* from a parental vector
Neo-F	CTCGAGTCAGAAGAACTCGTCAAGAAGGCG	To amplify *Neo* from a parental vector
Neo-R	GGGGAGATCTATGATTGAACAAGATGGATTGCACGC	To amplify *Neo* from a parental vector
ShCXCL12-F	CGGGCTTAGACTAAGGCCATTATTCTCGAGAATAATGGCCTTAGTCTAAGCTTTTTG	To form shCXCL12

ShCXCL12-R	AATTCAAAAAGCTTAGACTAAGGCCATTATTCTCGAGAATAATGGCCTTAGTCTAAGCCCGGTAC	oligoes following annealing
Transgene (ACTB promoter-GFP)-F	CGCCTCCGACCAGTGTTTGC	To detect the transgene contained in Envy EMSC in transplanted mice via PCR
Transgene (ACTB promoter-GFP)-R	TCGCCTTGATGCCGTTCTTCT	
GAPDH/Gapdh-F	ACCACAGTCCATGCCATCAC	To detect human GAPDH or mouse Gapdh as a loading control for RT-PCR
GAPDH/Gapdh-R	TCCACCACCCTGTTGCTGTA	
CXCR4-F	ACGCCACCAACAGTCAGAG	

CXCR4-R	AGTCGGGAATAGTCAGCAGGA	To detect CXCR4 expression via RT-PCR
Mouse Cxcr4-F	CTTCTGGGCAGTTGATGCCAT	To detect Cxcr4 expression via RT-PCR
Mouse Cxcr4-R	CTGTTGGTGGCGTGGACAAT	
iNOS-F	GTTCTCAGCCCAACAATACAAGA	To detect expression of markers for M1 macrophage via RT-PCR
iNOS-R	GTGGACGGGTCGATGTCAC	
IL-6-F	CTGCAAGAGACTTCCATCCAG	
IL-6-R	AGTGGTATAGACAGGTCTGTTGG	
IL-1β-F	GAAATGCCACCTTTTGACAGTG	
IL-1β-R	TGGATGCTCTCATCAGGACAG	
Argi-F	CTCCAAGCCAAAGTCCTTAGAG	

Argi-R	GGAGCTGTCATTAGGGACATCA	To detect expression of markers for M2 macrophage via RT-PCR
CD206-F	CTCTGTTCAGCTATTGGACGC	
CD206-R	TGGCACTCCCAAACATAATTTGA	
IL10-F	GCTGGACAACATACTGCTAACC	
IL10-R	ATTTCCGATAAGGCTTGGCAA	
hTK1-F	ATGCTGATGTCTGGGTAGGGTG	To detect *hTK1* gDNA in tissues isolated from EMSC-transplanted mice via PCR
hTK1-R	TGAGTCAGGAGCCAGCGTATG	

Supplemental Figures and Legends

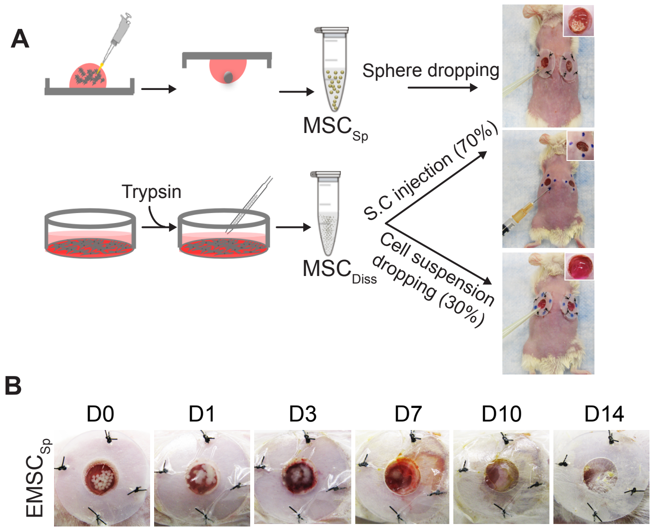

Figure S1. Topical application of MSC$_{Sp}$ or MSC$_{Diss}$ to skin wounds and wound healing post-MSC$_{Sp}$ transportation. Related to Fig.1.

(A) EMSC$_{Sp}$ were prepared using the hanging-drop method, and dropped onto wound surface (upper). EMSC$_{Diss}$ were prepared by dissociating EMSC with Trypsin, and 70% of the dissociated cells for a designated dose was injected into the skin surrounding the wound and 30% of them was mixed with Matrigel and topically applied to the wound surface (bottom).

(B) Representative images of wounds taken at various days post transplantation of EMSC$_{Sp}$.

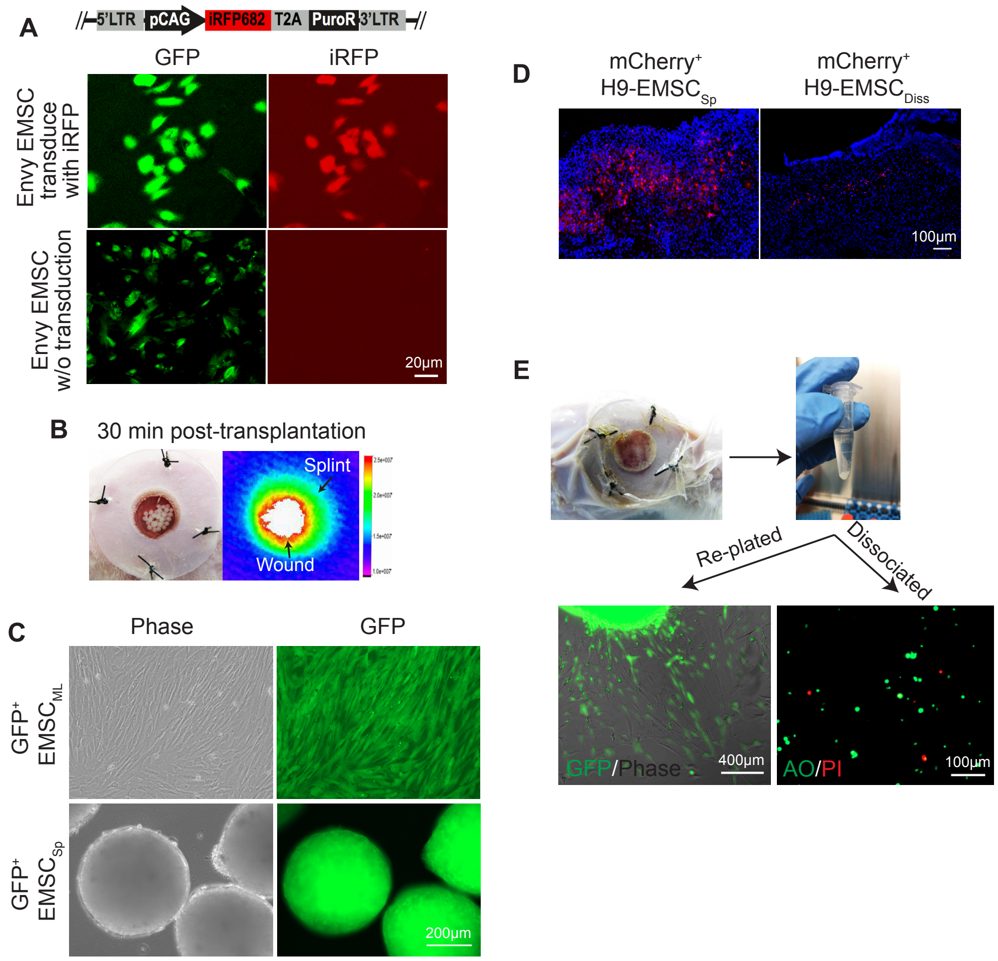

Figure S2. Viability and proliferation of transplanted EMSC and subsequent *ex vivo* culture.

(A) Envy hESC (GFP^{+}) were transduced with a lentiviral vector (upper) to ectopically express iRFP (bottom) for direct tracking of iRFP^{+} cells following transplantation.

(B) iRFP^{+} Envy EMSC$_{Sp}$ topically applied to a wound center (left) were fluorescently detected (right) under the In Vivo Xtreme System with the excitation filter at 630 nm

and emission filter at 700 nm. The color bar indicates the fluorescence intensity. The cells were used in Fig. 2A.

(C) EMSC derived from Envy hESC (GFP^+) were cultured either in monolayer ($EMSC_{ML}$) (upper) or spheres ($EMSC_{Sp}$) (bottom), and photographed in both bright field (left) and fluorescence (right).

(D) $mCherry^+$ H9 $EMSC_{Sp}$ or $EMSC_{Diss}$ were transplanted onto skin wounds, which were dissected at day 14 post-wounding, and cryosectioned for direct observation of $mCherry^+$ cells in the wounds.

(E) At day 4 following transplantation with Envy $EMSC_{Sp}$, the spheres that remained on the wound surface were flashed off with medium and precipitated. Some of the spheres were plated back to a culture dish, where a part of an attached and spreading sphere is shown. The rest spheres were dissociated for AO/PI staining to test the cell viability.

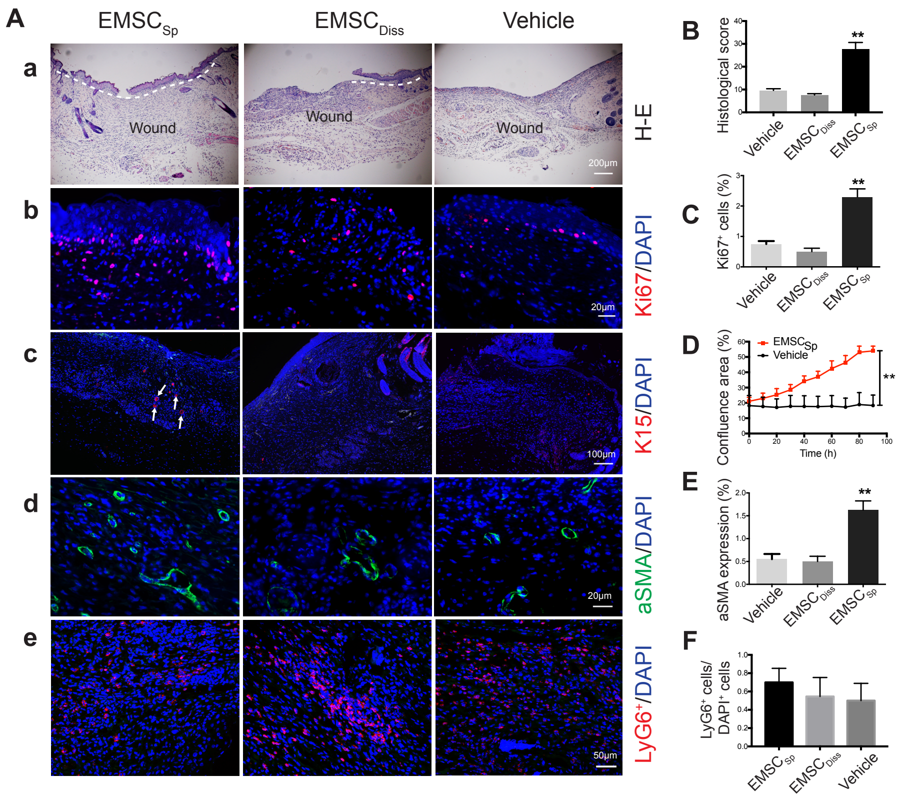

Figure S3. Analyses of EMSC$_{Sp}$-transplanted wounds.

(A) Histological examination of day-14 wounds transplanted with EMSC$_{Sp}$, EMSC$_{Diss}$ or vehicle control. H-E staining shows the extent of re-epithelialization of the wounds. Immunostaining for Ki67 (b), K15 (c), α-SMA (d) and LyG6 (e) indicates proliferating cells, hair follicle stem cells, smooth muscle cells, and granulocytes, respectively.

(B) The histological score for the H&E stained wounds in (A). n = 5 biological repeats; $^{**}P < 0.01$ for EMSC$_{Sp}$ versus EMSC$_{Diss}$ per Kruskal-Wallis test.

(C) Quantification of Ki67$^+$ cells in (A) n = 5 biological repeats; **$P < 0.01$ for $EMSC_{Sp}$ versus $EMSC_{Diss}$ or vehicle per Kruskal-Wallis test.

(D) Proliferative curves of dissociated cells from tissues isolated from day-7 wounds transplanted with $EMSC_{Sp}$ and vehicle control. The proliferation was monitored in the IncuCyte Real-time Imaging System. **$P < 0.01$ per Mann-Whitney U test.

(E) Quantification of α-SMA$^+$ cells in (A). n = 5 biological repeats; **$P < 0.01$ for $EMSC_{Sp}$ versus $EMSC_{Diss}$ or vehicle per Kruskal-Wallis test.

(F) Quantification of LyG6$^+$ cells in (A).

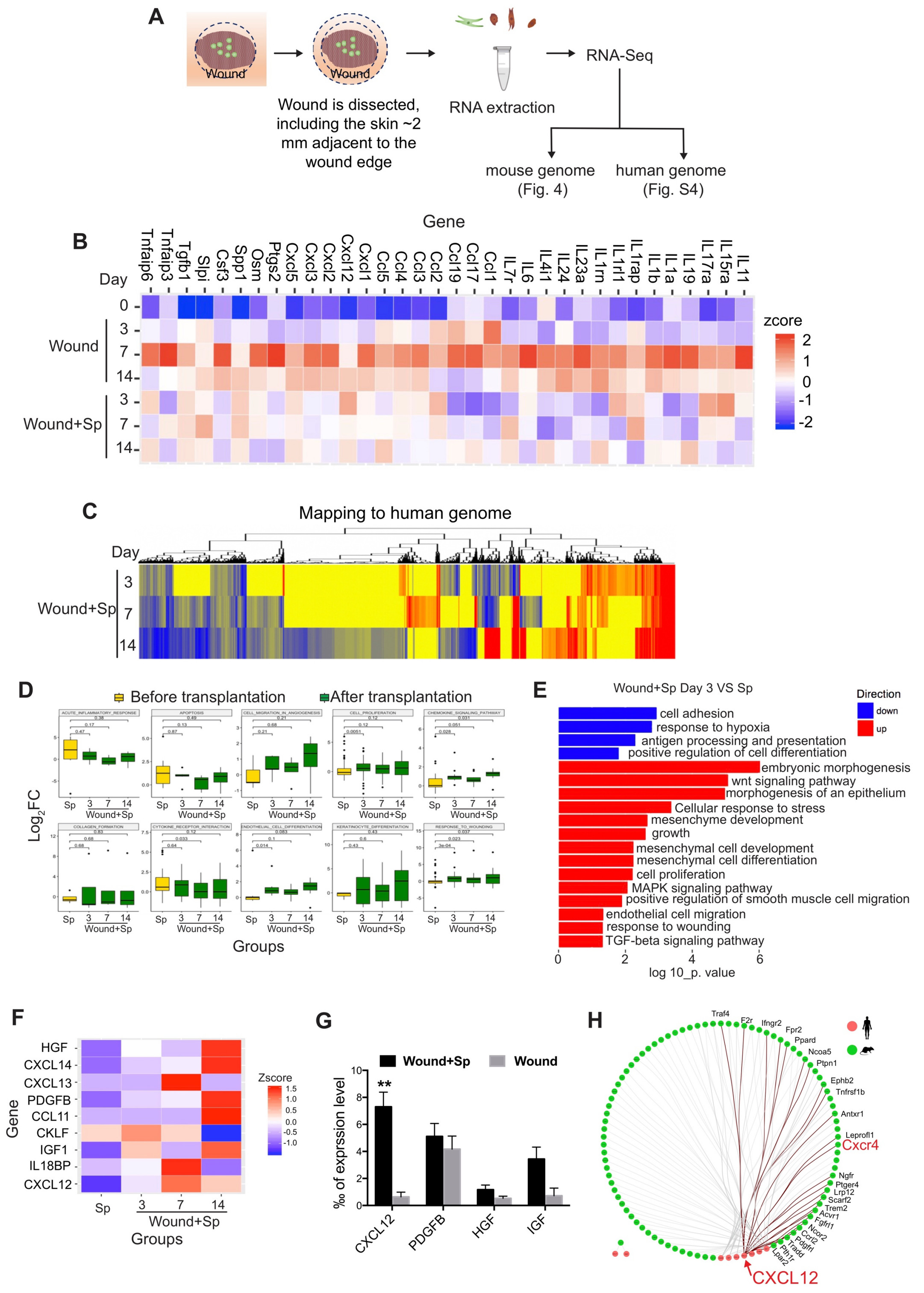

Figure S4. Transcriptomic analysis of EMSC before and after transplantation.

(A) Scheme for sample preparation, RNA-seq, and data analysis.

(B) Heatmap for expression of genes for interleukins, chemokines, and other inflammation regulators during healing of mouse skin wound alone (Wound) or treated with $EMSC_{Sp}$ (Wound+Sp) at 0, 3, 7, and 14 days post-treatment.

(C) Heatmap of differentially regulated human transcripts in $EMSC_{Sp}$-transplanted wounds (Wound+Sp) at days 3, 7, and 14 post-transplantation, compared with $EMSC_{Sp}$ before transplantation (Sp).

(D) Boxplots showing expressional changes of human genes associated with Gene Ontology-categorized eight signaling pathways or functional groups in the above samples. Each plot represents a pathway or functional group. The yellow boxes stand for $EMSC_{Sp}$ (Sp), and green for $EMSC_{Sp}$-transplanted wounds at different days post-transplantation.

(E) Enrichment analysis of human DEG in day-3 $EMSC_{Sp}$-transplanted wounds versus $EMSC_{Sp}$. The bar length represents −log10 *P* value. The red and blue bars represent terms enriched for up- and down-regulated genes, respectively.

(F) Heatmap showing the z-score value for expressional changes of human genes encoding secretable proteins in the above samples. The selected genes are differentially

expressed genes identified in at least one comparison between the three transplanted samples .

(G) Measurement of multiple cytokines in day-14 skin wounds treated with or without Envy (GFP^+) EMSC. n = 5 biological repeats. *$P < 0.05$ $EMSC_{Sp}$ versus $EMSC_{Diss}$ or Vehicle per ANOVA analysis.

(H) Pearson’s correlation coefficient between the differentially expressed genes in (F) and mouse receptor encoding genes identified from gene ontology. Red lines represent the correlation between human CXCL12 expression with their responding mouse receptors.

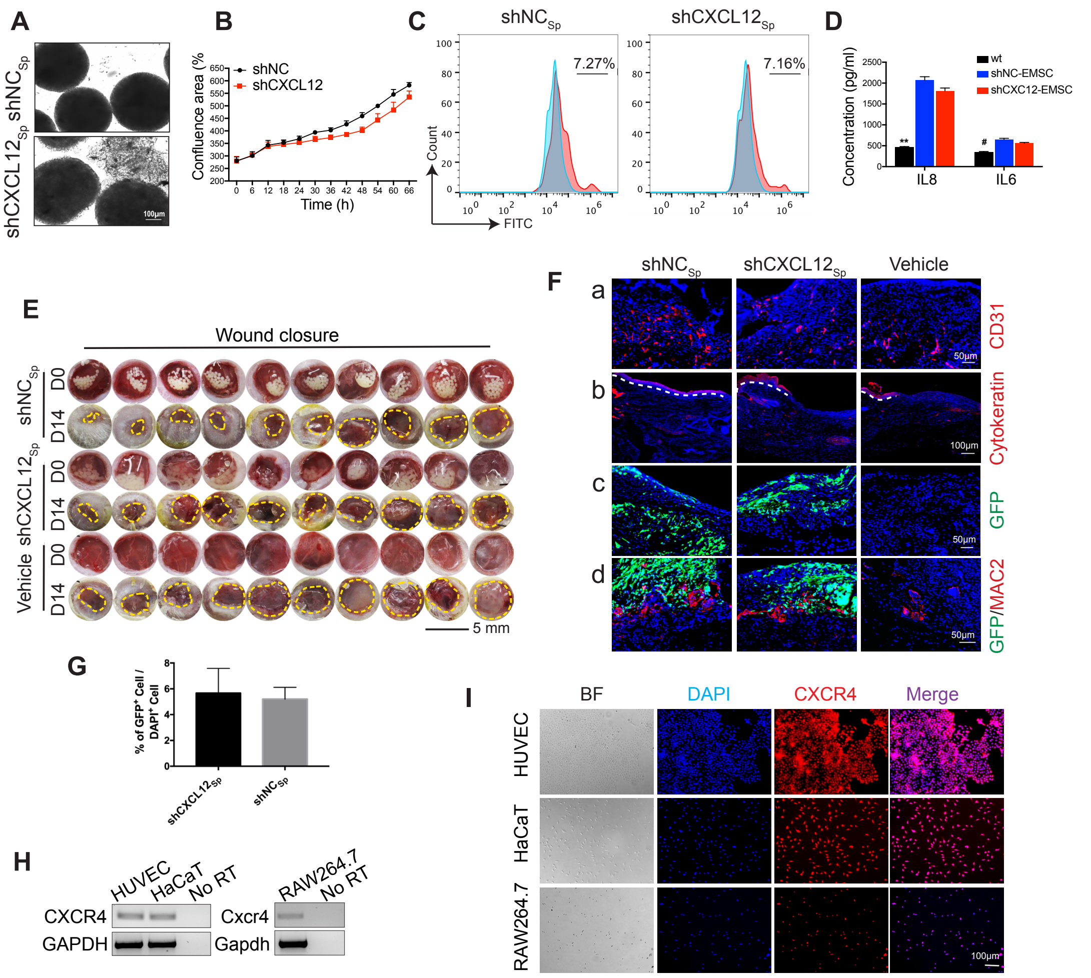

Figure S5. Association of repressed CXCL12/CXCR4 axis with impaired wound healing.

(A) Morphology of $EMSC_{Sp}$ including $shNC_{Sp}$ and $shCXCL12_{Sp}$ under phase contrast microscope.

(B) Prolifertion of dissociated $shNC_{Sp}$ and $shCXCL12_{Sp}$, monitored by the IncuCyte System.

(C) Flow cytometry for dead (PI^{+}) cells among dissociated $shNC_{Sp}$ and $shCXCL12_{Sp}$.

(D) Cytokine production by EMSC transduced with shCXCL12 or shNC and non-transduced control (wt). The same number (5×10^5/well) of shNC-EMSC, shCXCL12-EMSC, and wild-type EMSC were seeded in triplicate and cultured in αMEM medium. After 48 hours, the conditioned medium from each group was collected and processed for CBA analysis. $^{**}P < 0.01$ for wt EMSC versus shNC-EMSC and shCXCL12-EMSC. #$P < 0.05$ for wt EMSC versus shNC-EMSC per two-way ANOVA analysis.

(E) Images of wounds transplanted with $shNC_{Sp}$ and $shCXCL12_{Sp}$ at days 0 and 14 post-transplantation. Unclosed wound areas at day 14 are marked with yellow dashed lines.

(F) Histological examination of day-14 wounds transplanted with $shNC_{Sp}$ and $shCXCL12_{Sp}$ or vehicle. Immunostaining for CD31 (a), cytokeratin (b), GFP (c) and MAC2 (d), indicates vascular endothelial cells, epithelial cells, engrafted EMSC, and macrophage, respectively.

(G) Percentage of GPF^+ cells among cells isolated from skin wound 14 days after transplantation with $shNC_{Sp}$ or $shCXCL12_{Sp}$.

(H) Detection of CXCR4 expression in HUVEC and HaCaT and Cxcr4 in murine RAW264.7 cells via RT-PCR. The primers were designed and listed in Table S2. No RT stands for no reverse transcriptase used during reverse transcription of the corresponding mRNA.

(I) CXCR4 in HUVEC, HaCaT, and RAW264.7 cells was detected via immunostaining.

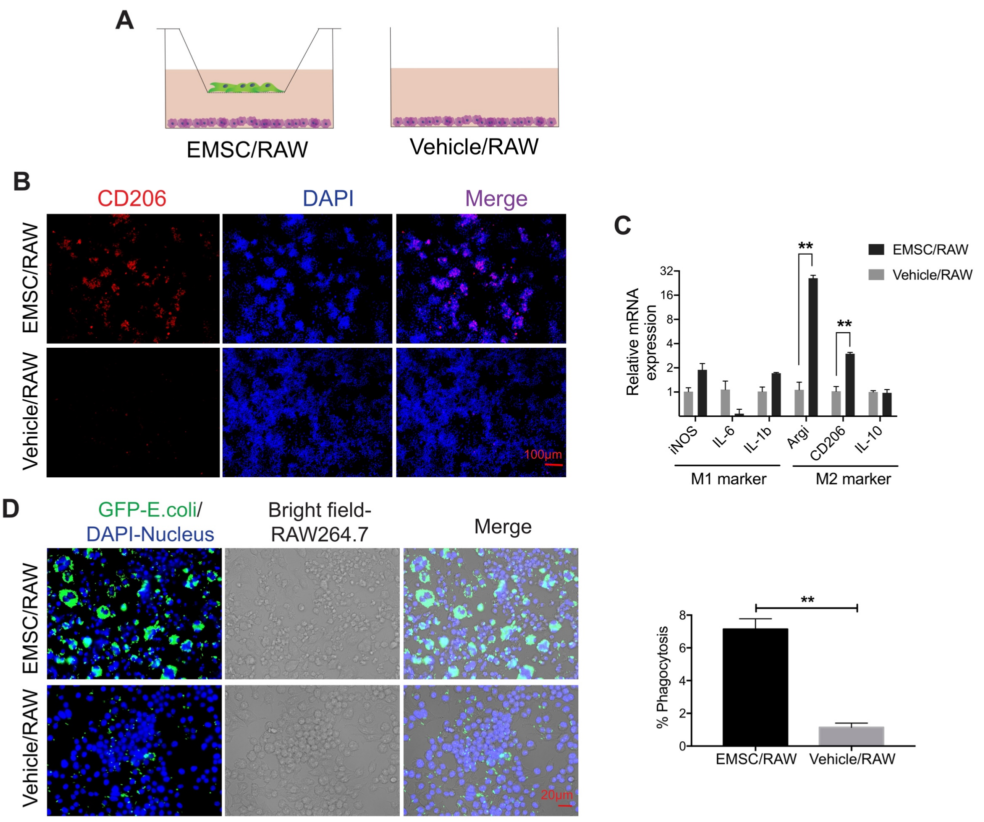

Figure S6. EMSC promote polarization and phagocytosis of murine macrophage in a co-culture system.

(A) Mouse RAW264.7 macrophages were seeded in the lower compartment of a Transwell and EMSC seeded in the upper insert above a filter (EMSC/RAW). The macrophage cultured with the vehicle only (Vehicle/RAW) serves as a control.

(B) Following the co-culture for 48 hours, the macrophages in the lower compartment were immunostained for the M2 macrophage marker CD206 and counterstained with DAPI for the nuclei.

(C) Some of the macrophages from A were tested via qPCR for expression of M1 macrophage markers *iNOS*, *IL6*, and *IL1b* and M2 markers *Argi*, *CD206*, and *IL10*. $^{**}P < 0.01$ per Student's *t*-test.

(D) Alternatively, some of the macrophage from A were incubated with GFP-labeled E. coli bioparticles. After 1-hour incubation, the cells were fixed and stained for GFP (E. coli) and counterstained with DAPI. Representative images are shown for macrophage phagocytosis under high magnification and displayed as % of phagocytosis (see Supplemental Materials and Methods). $^{**}P < 0.01$ per Student's *t*-test.

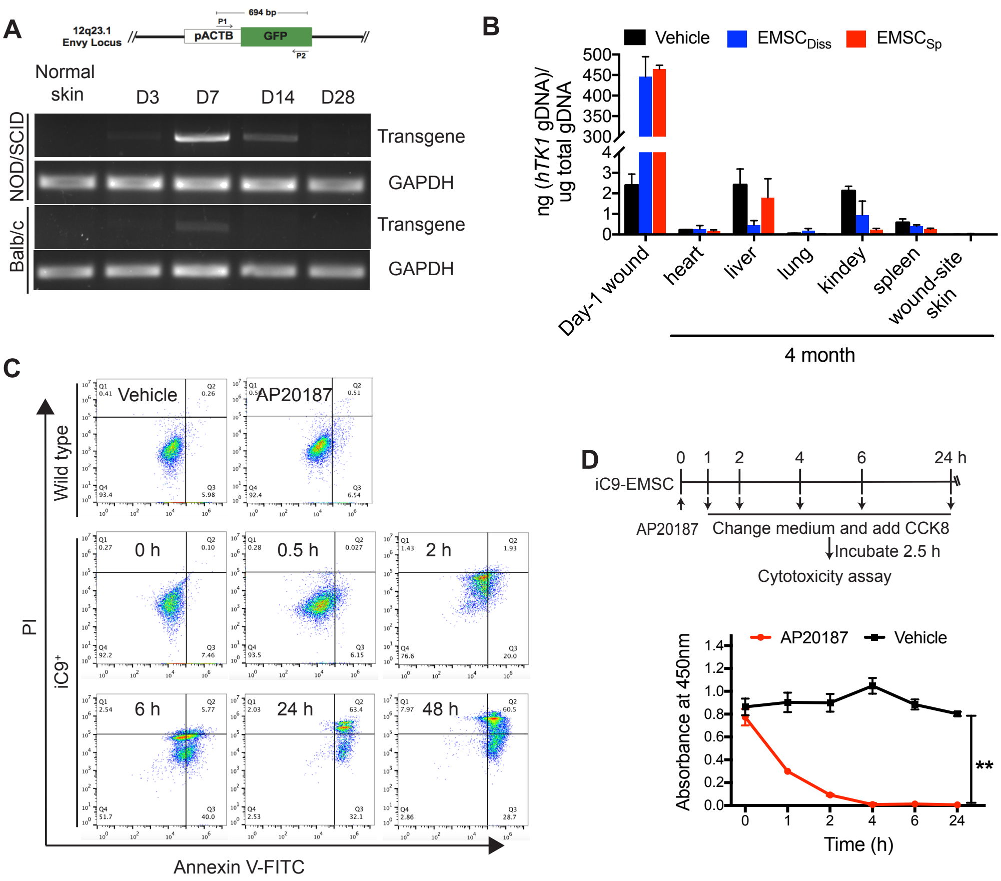

Figure S7. Long-term tracking of EMSC DNA in $EMSC_{Sp}$-transplanted wound, and AP20187-induced death of iC9-expressing EMSC *in vitro*.

(A) Detection of Envy EMSC DNA in $EMSC_{Sp}$-transplanted wounds via PCR. The primers were designed for amplification of a part of the transgene including the *ACTB* promoter and *GFP* in Envy EMSC (upper). Results are shown (bottom) with gDNA as templates isolated from wounds at various times post-transplantation with $EMSC_{Sp}$ in NOD/SCID and Balb/c mice. Normal skin without EMSC transplantation was used as a negative control, and a fragment of GAPDH DNA detected as a loading control.

(B) Detection of *hTK1* gDNA in mice four months after transplantation with 1×10^6 EMSC (as dissociated cells or in spheres) onto skin wound. The starting control "Day-1 wound" refers to gDNA isolated from the wound at day 1 after the transplantation with $EMSC_{Sp}$ or vehicle control.

(C) Representative histograms showing flow cytometry results for apoptotic cells (related to Fig. 6C). No marked difference was observed between wild-type H9 EMSC pre-treated with and without AP20187 for 24 hours (upper). In contrast, apoptosis developed in iC9-expressing EMSC during exposure to the AP20187 for various times (bottom). Annexin V^-/PI^-, Annexin V^+/PI^-, and Annexin V^+/PI^+, and Annexin V^-/PI^+ represent live, early apoptotic, late apoptotic, and dead cells, respectively.

(D) Cytotoxicity assay on iC9-expressing EMSC treated with or without AP20187 for various times as shown in the scheme (upper). Live cells were determined using the Cell Counting Kit-8 (Beyotime) by detecting the color absorbance at 450 nm (bottom). $^{**}P < 0.01$ per Student's *t*-test.

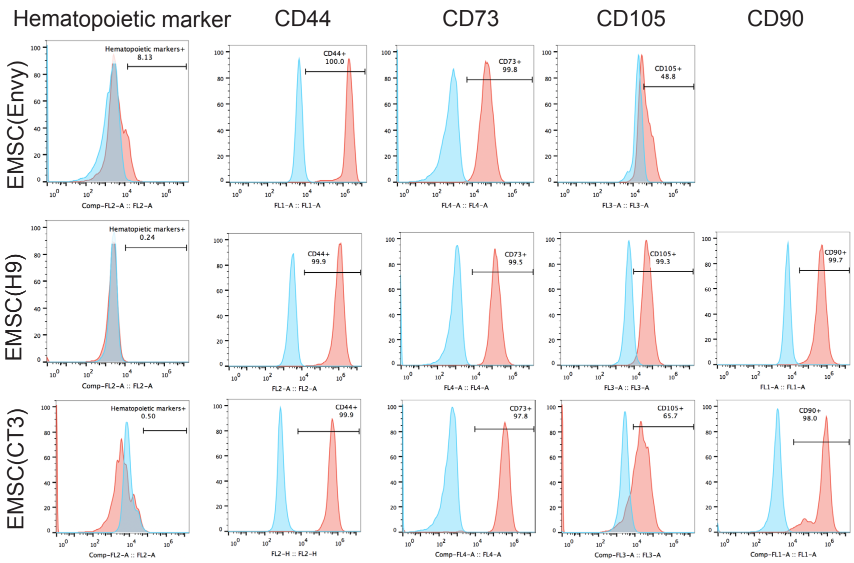

Figure S8. Characterization for cell surface markers on EMSC lines used in this study via flow cytometry.

The blue peaks represent isotype controls and red peaks depict the expression of the cell surface markers. Since Envy hESC-derived EMSC were GFP^{+}, they were not suitable for detection of CD90 could not be tested as the antibody used the green fluorescent channel FITC.

Reference

1. Ambrosio AL, Boyle JA, Aradi AE, Christian KA, Di Pietro SM. TPC2 controls pigmentation by regulating melanosome pH and size. Proc Natl Acad Sci U S A. 2016; 113: 5622-7.

2. Hong AL, Tseng YY, Cowley GS, Jonas O, Cheah JH, Kynnap BD, et al. Integrated genetic and pharmacologic interrogation of rare cancers. Nat Commun. 2016; 7: 11987.

3. Wu Y, Chen L, Scott PG, Tredget EE. Mesenchymal stem cells enhance wound healing through differentiation and angiogenesis. Stem Cells. 2007; 25: 2648-59.

4. Song P, Xie Z, Guo L, Wang C, Xie W, Wu Y. Human genome-specific real-time PCR method for sensitive detection and reproducible quantitation of human cells in mice. Stem Cell Rev. 2012; 8: 1155-62.
